# Considerations of equity in the development of tools that identify and respond to end-of-life carer support needs: a scoping review

**DOI:** 10.1136/bmjopen-2026-121897

**Published:** 2026-09-11

**Authors:** Hannah A Long, Christine Rowland, James Higgerson, Alex Hall, Sean Urwin, Joanna Brooks

**Affiliations:** 1 British Columbia Centre on Substance Use, Vancouver, British Columbia, Canada; 2 Department of Medicine, The University of British Columbia, Vancouver, British Columbia, Canada; 3 Manchester Centre for Health Psychology, University of Manchester, Manchester, UK; 4 School of Health Sciences, University of Manchester, Manchester, UK

**Keywords:** PALLIATIVE CARE, Caregivers, Review

## Abstract

**Abstract:**

**Objectives:**

To (1) systematically identify tools developed to assess and address the support needs of informal carers in palliative or end-of-life care and (2) examine the extent to which socially stratifying sample characteristics have been reported following tool use.

**Design:**

A systematic scoping review was conducted.

**Methods:**

Medline, CINAHL, PsycINFO, ASSIA, ProQuest Central and Web of Science databases were searched from inception to May 2024 and again in May 2025. Primary studies using any methodology reporting the development and/or use of tools to assess informal carer support needs in end-of-life care were included. Consistent with scoping review methodology, we did not conduct a quality appraisal of included studies. We extracted tool characteristics, frequency of use and sample characteristics according to PROGRESS+.

**Results:**

A total of 74 articles were included. From these, 24 unique tools were identified. Nine tools were used in ≥2 articles, while 15 were used only once. The most commonly used tools were the Carer Support Needs Assessment Tool (35 articles), the Family Inventory of Needs (6 articles), the Carer Alert Thermometer (5 articles) and the Spiritual Needs Inventory (4 articles). There was limited evaluation of tools beyond psychometric validation. There were substantial gaps in equity-informed caregiver sample reporting. While gender, age and country of participants were commonly reported, most other PROGRESS+characteristics were inconsistently or minimally reported.

**Conclusions:**

While numerous tools exist to assess and address informal caregiver support needs, there is considerable variation in tool uptake, evaluation and the extent to which tools consider equity factors. There is a need for equity-informed tool development and clearer conceptualisation of caregiver needs, alongside efforts to evaluate tool implementation, impact and sustainability in palliative care settings.

**STRENGTHS AND LIMITATIONS OF THIS STUDY**

- This scoping review uses a systematic and transparent approach to identify and synthesise literature on the development of tools assessing informal caregiver support needs, including consideration of equity.
- We used a recognised equity framework (PROGRESS+) to record equity characteristics for both carer and patient samples.
- We used a comprehensive search strategy, including six databases, updated searches and reference searching, to identify and describe the literature. The article is reported according to the PRISMA extension for scoping reviews.
- We did not search for grey literature and we excluded articles written in languages other than English. There may be additional relevant articles that were not identified.
- It was beyond the scope of this review to examine the psychometric properties of the identified tools and to consider equity across the tools’ lifecycles (ie, equity in tool design, development, application and evaluation).

## Introduction

 Most patients receiving palliative and end-of-life care would prefer to be cared for at home.^1 2^ As a result, family and friend caregivers (often referred to as unpaid or informal caregivers) play a critical role in supporting patients during this period. Informal caregivers primarily provide free labour, generating substantial economic value and significant savings for health and social care systems.^3 4^ Although caregiving can be experienced as meaningful and fulfilling, many carers face high physical, practical and emotional demands in their role, resulting in considerable personal, financial and health-related burdens.^5–8^

While palliative care has long endorsed a ‘unit of care’ approach that includes families, health service provision has mostly prioritised patient needs over those of caregivers.^9 10^ Consequently, caregivers often have unmet needs across practical, informational, emotional, social and financial domains—defined as the difference between services received and those perceived as necessary by the caregiver. Caregivers represent a highly heterogeneous group, varying across characteristics such as age, gender, relationship to the care recipient, cultural and socioeconomic background, health status and caregiving context, all of which may shape both caregiving experiences and support needs. There has been increasing recognition of carers as individuals with distinct (specific and actionable) support needs, alongside the development and implementation of tools intended to routinely identify and systematically assess and address these needs within healthcare and social care practice.^11^

There is well-established evidence of health inequities in palliative and end-of-life care,^12^ documented across multiple sociodemographic characteristics. Poorer quality end-of-life care has been reported among people from minoritised ethnic communities, those living in deprived or rural areas and individuals with non-malignant conditions.^12^ Research in palliative and end-of-life care has been criticised for the underrepresentation of diverse populations, raising calls to improve equity and representativeness in both research and practice.^13–15^ A recent scoping review further highlighted the importance of understanding how overlapping identities, characteristics and social positions may interact to compound disadvantage and demonstrated the lack of existing research using intersectionality theory to understand access to, use and experience of palliative care.^13^

Despite this growing attention on inequities, little is known about how carer support needs are assessed across different social groups in end-of-life care. While some evidence suggests that certain groups of caregivers’ experience distinct or inadequately met needs,^16^ even less is known about whether carer needs assessment tools account for socially stratifying factors in their development, use or evaluation. This reflects a wider methodological gap, not only in the carer literature^11^ but in clinical and health outcome measurement across research fields (eg, cancer,^17^ allergy and immunology^18^ and rheumatology^19^), where tools have often been developed and validated without explicit attention to equity. A recent narrative review indicated that carer support varies according to contextual factors such as cultural norms and cultural sensitivity of services and providers.^20^ As carer needs assessment tools are increasingly used in practice, there is a risk that tools developed without an explicit equity lens may exacerbate existing disparities.^21^

The present scoping review aimed to systematically identify tools developed to assess and address the support needs of informal carers providing end-of-life care and to examine whether and how potentially socially stratifying sample characteristics have been considered in the use of these tools. The review had the following research questions:

1. What tools have been developed to identify and respond to caregiver support needs in palliative and end-of-life care settings and what are the tools’ intended purpose?
2. What evaluation approaches have been used to assess these tools?
3. With whom have these tools been developed, and to what extent is equity considered in tool development?

## Methods

### Design

A scoping review protocol is available^22^ (online supplemental materials 1). The review was conducted in accordance with the Joanna Briggs Institute methodology for scoping reviews^23^ and Arksey’s and O’Malley’s methodological framework for scoping reviews.^24^ This article is reported according to the Preferred Reporting Items for Systematic Reviews and Meta-Analyses (PRISMA) extension for scoping reviews (online supplemental materials 3). The review involved four sequential stages: searches, screening, data extraction and data charting.

### Searches

Two reviewers (HAL, JB) conducted searches in the electronic databases Medline (Ovid), CINAHL (EBSCO), PsycINFO (Ovid), ASSIA (ProQuest), ProQuest Central (ProQuest) and Web of Science Core Collection from inception to 15 May 2024. The searches were updated on 21 May 2025. The search strategy was tailored to each database, including all identified thesaurus terms, keywords and free-text terms. Searches were limited to English-language articles, excluded case reports, editorials, letters and opinion pieces and excluded articles for which the full text was unavailable. We did not exclude articles based on publication status. We conducted forward citation searches using Google Scholar and hand searches of the included articles’ reference lists. The search strings for each database are available in online supplemental materials 4.

All articles were imported into EndNote reference management software. Duplicates were removed. The remaining articles were uploaded to Rayyan (www.rayyan.ai) to facilitate article screening by multiple reviewers. At least two reviewers screened all titles and abstracts and classified these as ‘include’, ‘exclude’ and ‘unsure’ (82% agreement). All conflicts were resolved through discussion between two or more reviewers until agreement was reached. At least two reviewers read the full texts of all potentially relevant articles against the eligibility criteria. Agreement was reached through discussion by two or more reviewers. Reviewers were not involved in the screening or data extraction of any studies to which they had contributed. Later interpretation of findings was conducted through discussion within the full research team, ensuring transparency, critical input from multiple researchers and minimising of the influence of any individual perspective.

### Eligibility criteria

We used the Population, Concept, Context framework to define our inclusion and exclusion criteria (summarised in table 1; further detail is available in the protocol^22^). We included articles reporting the development and use of tools to assess the support needs of carers of patients receiving end-of-life care. Drawing on previous work, particularly that of the developers of the Carer Support Needs Assessment Tool (CSNAT) (eg,^25^), our definition of a ‘needs assessment’ was a tool that provided direct information about a carer’s actionable help and support requirements (rather than tools to assess broader or less well-defined strains or problems, such as caregiver experience, caregiver satisfaction or other more indirect indicators of need). While we distinguished between caregiver support needs and other constructs (such as burden, strain and preparedness) and excluded studies using tools that measured these other constructs alone, we included measures where studies explicitly used them to identify or respond to carers’ support needs or where items were interpreted as indicating actionable areas for support.

**Table 1:** Eligibility criteria

PCC	Definition	Inclusion	Exclusion
Population	Informal carers of patients receiving palliative or end-of-life care.	Adult family and friend (informal) carers of adult patients who are receiving palliative and end-of-life care. Studies reporting on the implementation of a tool (which may include implementation studies where study participants are not informal carers)*	Studies of child patients or carers.
Concept	Tools or measures developed to assess the support needs of informal carers of patients receiving end-of-life care. Support needs encompass any practical, financial and emotional support to enable carers to provide care, plus direct personal support for carers themselves.	Studies that: Use a tool or measure to assess the current support needs of carers providing support to adults receiving palliative and end-of-life care. Report the development, validation, extension and translation of tools or measures to assess the support needs of carers providing support to adults receiving palliative and end-of-life care. Evaluate the implementation of tools or measures to assess carer support needs in palliative and end-of-life care.	Studies that: Assess patient (not carer) support needs, assess both but do not allow extraction of carer-specific data. Identify carer support needs without a formal assessment tool or the way support needs were identified is not reported (eg, qualitative studies reporting qualitative data on carer needs without use of a tool to assess these needs). An exception to this is studies underpinning the development of eligible tools. Assess future or prospective carer needs. Assess non-palliative and non-end-of-life contexts. Assess support needs of bereaved carers only or retrospectively assess carer support needs following a death. Assess carers’ psychological states, including emotional or mood states or well-being (eg, measures of anxiety and depression), which are not designed to identify the practical and emotional support carers need. Include measures to aid end-of-life care planning and decision-making, rather than carer support needs. Rely exclusively on a proxy or professional report of carer needs, without direct carer input to report their needs.
Context	Healthcare, social care or community setting.	Studies in any healthcare, social care or community setting in any country.	Studies not in a healthcare, social care or community setting

* Amendment from published protocol to allow for inclusion of relevant development work. These papers are not included in the data synthesis of PROGRESS+criteria, which relate only to carers and care recipients.

PCC, Population, Concept, Context.

### Data extraction

Data were extracted from included papers in two stages. In stage 1, one reviewer extracted data from all eligible articles using a standardised extraction sheet (published with the protocol^22^). This included details on the primary study (eg, authors, publication year, country, setting of study, study design, participants and sample size, outcome variables and justifications for choice of outcome variables) and the tool used to assess carer support needs (eg, tool description and study authors’ rationale for using the tool if provided). A second reviewer extracted data in stage 1 for 20% (k=25; where ‘k’ denotes the number of articles) of the eligible articles. Reviewer extractions were compared. Discrepancies were resolved through discussion with the full review team.

In stage 2, we used PROGRESS+^21^—an acronym referring to socially stratifying details (eg, place of residence; race/ethnicity/culture/language; occupation; gender/sex; religion; education; socioeconomic status; social capital plus other context-specific factors such as age, health condition and features of relationship)—to identify and extract sample characteristics associated with equity in studies with carer participants. This follows PRISMA-Equity guidance for reporting equity-focused reviews.^26^ For the purposes of this review, PROGRESS+factors were applied to both caregivers and patients. This reflects the relational nature of caregiving in palliative and end-of-life care, where patient characteristics (eg, diagnosis, disease trajectory and sociodemographic context) shape caregiving roles, experiences and associated support needs. Two reviewers extracted data from eligible articles. Disagreements between reviewers were resolved through discussion and with the input of additional reviewers, as needed.

### Data collation and charting

To answer the first research question, we tabulated all tools used in the eligible articles to assess and address the support needs of informal carers providing end-of-life care. Data on tool characteristics and intended purpose were extracted from each article based on descriptions provided by study authors. Categories relating to tool purpose and content were developed iteratively during the data extraction process through discussion within the research team. Categorisation was guided by how tools were described in the original articles; for example, where authors explicitly identified a tool as a screening instrument, it was classified as such. Tools comprising multiple domains were categorised as multi-domain, while those producing a single overall score were categorised as unidimensional. In cases where domain structure was not explicitly reported, categorisation was based on descriptions of the tool’s structure and content. Where possible, we extracted tool characteristics (eg, response options, number of items, scoring, administration details) and totalled the frequency of use. To ensure completeness of information for the most commonly used tools (used in ≥4 articles), we identified the original articles reporting the tools’ characteristics and extracted and summarised further tool details (eg, their original intended purpose). One tool was used in three articles but showed heterogeneity in its use across these articles. Tools used in ≤2 articles were considered to be used too infrequently to warrant closer attention.

To answer the second research question, we tabulated any outcome measures and evaluation approaches used to assess the validity, reliability and/or usability of these tools, including how frequently these approaches were used across the eligible articles. Findings from articles reporting evaluation work were categorised into one or more of the following: *validation* (assessing psychometric properties and whether the tool was perceived to assess what it was theorised to assess), *carer feedback* (studies providing feedback data on use of tool from carers), *practitioner feedback* (studies providing feedback data on use of tool from practitioners), *carer impact* (assessing carer outcomes as a result of tool use), *adoption* (studies reporting on implementation of the tool in a new population or context) and *descriptive only* (studies reporting descriptive data only, from tool use). Some articles reported more than one type of evaluation (eg, both carer feedback and practitioner feedback) and were thus counted in both categories.

To answer the third research question, findings were summarised descriptively and tabulated according to the PROGRESS+characteristics.

### Quality appraisal

Consistent with scoping review methodology, we did not perform a quality appraisal of the included studies. The focus of this review was to identify and map existing caregiver support needs assessment tools, patterns of outcome measurement and the reporting of socially stratifying participant characteristics in the literature to date.

## Results

### Searches

The electronic database searches identified a total of 476 records (326 after duplicates were removed). After title and abstract screening, 95 articles were read in full. A total of 58 articles were eligible for inclusion. The updated searches identified a total of 40 articles (34 after duplicates were removed). On inspection, a further four articles were removed, as these were identified by the original searches. Of the remaining 30 articles, 6 were considered potentially relevant, and the full texts were read. Of the six, three were eligible. Forward citation and hand searching of all eligible articles identified a further 22 potentially relevant articles. All texts were read in full. Of these, 13 were eligible. Therefore, in total, 74 articles were included in this scoping review. See online supplemental materials 2 for a PRISMA flow diagram and article references.

### Article characteristics

The 74 articles were published across four decades, from the 1990s (k=2), the 2000s (k=7), the 2010s (k=34), to the 2020s (up to 2025; k=31), reporting research in the following countries: the UK (k=20), Australia (k=12), Canada (k=5), China (k=6; of which k=4 are published in Hong Kong), the US (k=8), the Netherlands (k=4), Sweden (k=4), Austria (k=3), Denmark (k=3), the Czech Republic (k=2), Germany (k=3), Switzerland (k=1), Japan (k=1), Bhutan (k=1), Ireland (k=1), Singapore (k=1), Indonesia (k=1), Taiwan (k=1), Iceland (k=1) and Turkey (k=1) (some papers report on research undertaken in more than one country). See online supplemental materials 7 for a table of included articles.

#### RQ 1: What tools have been used to assess carer-reported support needs in end-of-life or palliative care settings, and what is the intended purpose of these tools?

Across the 74 included articles, a total of 24 unique tools were used to assess carer-related support needs. The identified tools predominantly focused on identifying caregiver needs, with a smaller number designed primarily for evaluation or outcome measurement. There was substantial variation in scope, ranging from tools focused on a single domain (eg, spiritual needs) to multi-domain instruments intended to support broader, more holistic assessment of caregiver support needs. Nine tools were used in ≥2 articles (see table 2), while 15 tools were used in a single article. The most frequently used tools (≥4 articles) were the CSNAT (k=35), the Family Inventory of Needs (FIN) (k=7), the Carer Alert Thermometer (k=5) and the Spiritual Needs Inventory (SNI) (k=4). A brief description is given for these, using details extracted from three included articles reporting the original tools’ development^22–24^ and a fourth article reporting the SNI,^25^ originally developed with patients rather than carers. Details on the 24 tools are provided in online supplemental materials 5.

**Table 2:** Tools used in ≥2 articles

Tool name	Number of articles using tool
Carers Alert Thermometer (CAT)	5
Carer Support Needs Assessment Tool (CSNAT/CSNAT-I)	35
Caregiver Strain Index (CSI)	2
Family Inventory of Needs (FIN)	7
Home Carer Needs Survey (HCNS)	2
Needs Assessment Tool-Progressive Disease (NAT-PD)*	3
Spiritual Needs Inventory (SNI)	4
Family Satisfaction with End-of-Life Care tool (FAMCARE)	2
Problems and Needs in Palliative Care Questionnaire (PNPC)	2

* Cancer and heart failure versions are included and an adapted version for interstitial lung disease.

##### Carer support needs assessment tool

The Carer Support Needs Assessment Tool (CSNAT, now known as the CSNAT-I (intervention)) was developed in the UK. The first article reporting the development of the CSNAT (a qualitative study^27^ with bereaved carers identifying key areas of support) was published in 2013, followed by a validation study with current carers.^25^ The tool originally comprised 14 broad support domains: 7 relating to support enabling the carer to care for the patient and 7 relating to the carer’s own well-being. A further domain addressing support needs related to relationship changes was added in 2020.^28^ The tool is intended to allow carers to identify, express and prioritise areas (domains) in which they need more support. This is followed by a needs-led conversation with a practitioner to explore the carer’s individual needs and identify appropriate support to meet those needs.

##### Family inventory of needs

The FIN was developed in Canada for use with caregivers of patients with advanced cancer. An article reporting the development, reliability and validity of the instrument was published in 1995.^29^ The tool consists of 20 items, each rated on two subscales: the importance of the need and the extent to which the need is perceived to be met. The tool is intended to assess family caregiving needs in the context of advanced cancer, using ratings of importance and need fulfilment to identify unmet needs that may require support or intervention.

##### Carers alert thermometer

The Carers Alert Thermometer (CAT) was developed in England by Knighting *et al*.^30^ The original tool contained 10 questions across eight domains that can be grouped into two overarching themes: current caring needs and carers' health and well-being. The tool is intended to be used as an alert indicator to identify carers who may be at risk of strain or have unidentified and unmet support needs.

##### Spiritual needs inventory

The SNI was developed by Herman^31^ in the US and is intended to assess the spiritual needs of end-of-life patients. The tool contains 17 items that cover 5 domains: outlook, inspiration, spiritual activities, religion and community.

### RQ 2: What evaluation approaches have been used to assess these tools?

Of the 24 tools identified, 14 have undergone some type of evaluation, including *validation* (10 tools in k=24), *practitioner feedback* (4 tools in k=12), *carer feedback* (4 tools in k=10), *carer impact* (4 tools in k=7) *and adoption* (1 tool in k=1). Across categories, the CSNAT-I has been evaluated most extensively (k=30), followed by the CAT (k=5) and the FIN (k=4). Online supplemental materials 5 provides additional detail on the types of evaluation used per tool.

### RQ3: With whom have these tools been developed? To what extent is equity considered in tool development?

Of the 74 articles included in the full data extraction, 64 articles included samples of informal caregivers, while the remaining papers (k=10) were limited to samples of healthcare practitioners and/or healthcare volunteers. Of the 64 articles involving informal caregivers, it was possible to clearly discern the country of residence for all articles even if this was not explicitly reported. Most articles reported caregiver gender (k=56, 87.5%), age (k=56, 87.5%) and relationship between caregiver and care recipient (k=49, 76.6%). Around two-thirds of the articles reported caregiver education (k=44 68.75%). Some information on caregiver occupation (though often very broadly defined) was reported by k=37 (57.8%). All other PROGRESS+characteristics were reported by fewer than half of the included studies (table 3). We were unable to report on ‘other details of residence’ as articles reported this in terms of study setting (eg, hospice, home care) relating to the care recipient rather than the carer. The main findings are summarised in table 3 and reported in more detail in online supplemental materials 6.

**Table 3:** Summary of sample characteristics according to PROGRESS+

Characteristic	Articles (k)	Reported Y (%)	Reported N (%)	Overall findings
Country of residence*	64	64 (100.0%)	–	19 countries represented. The largest number of studies were conducted in the UK (k=11), Australia (k=10), the US (k=8), China/Hong Kong (k=6) and Canada (k=5).
Ethnicity/race (carer)	64	22 (34.4%)	42 (65.6%)	When reported, participants were predominantly Caucasian and non-Hispanic white.
Ethnicity/race (care recipient)	64	2 (3.1%)	62 (96.9%)	This was generally not reported.
Occupation† (caregiver)	64	37 (57.8%)	27 (42.2%)	Typically reported as a broad employment status (employed, unemployed, retired, etc.) with limited detail on occupational role or class.
Gender (caregiver)	64	56 (87.5%)	8 (12.5%)	Most (k=53) reported a higher proportion of female caregivers.
Gender (care recipient)	64	21 (32.8%)	43 (67.2%)	Male patients were more frequently represented than female patients (k=13).
Religion	64	8 (12.5%)	56 (87.5%)	When reported, participants were predominantly Christian.
Education	64	44 (68.75%)	20 (31.25%)	Samples were generally well educated, with most participants having at least secondary education and many having post-secondary qualifications.
Socio-economic status	64	17 (26.6%)	47 (73.4%)	When reported, income categories were typically reported, with samples mainly including participants reported as being from the lowest or second-to-lowest income group.
Caregiver age	64	56 (87.5%)	8 (12.5%)	Caregivers were predominantly middle- to older-aged adults.
Care recipient age	64	24 (37.5%)	40 (62.5%)	Care recipients were predominantly in the older adult range overall, but with samples spanning from early adulthood to very old age.
Relationship between caregiver and care recipient	64	49 (76.6%)	15 (23.4%)	The most common caregiver relationship was spousal/partner (k=36), followed by adult children (k=13).
Patient’s condition	64	41 (64.1%)	23 (35.9%)	Most studies (k=30; 73% of the papers reporting this information) focused exclusively or primarily on cancer populations, with some limited inclusion of non-malignant conditions.
Any information on carer health condition	64	5 (7.8%)	59 (92.2%)	Carer health was largely unreported. Only k=1 recorded any specific carer health conditions. A further k=4 recorded more general information on carer health status.

* For country of residence, although not all papers reported this explicitly, where authoring teams were based in a single country and/or referenced specific national healthcare systems, it was clearly evident where the research was undertaken, and so the country of residence was coded as having been reported.

† Reported N (%) reflects whether a characteristic was reported in each study but does not capture the level, consistency or granularity of reporting (eg, broad categories such as ‘employed/retired’ vs more detailed classifications).

## Discussion

This scoping review identified 74 articles and 24 unique tools developed to assess and address the support needs of informal carers in palliative or end-of-life care. Our findings indicate three main patterns. First, one tool in particular (the CSNAT-I) dominates this field, featuring in almost half of all identified articles and approximately six times more than the second most used tool (the FIN). Two-thirds of the identified tools appeared only once in the literature. Second, there was limited evaluation of tools beyond psychometric validation. Third, there are substantial gaps in the consideration of equity-related characteristics in tool development. While country of residence, gender and age were commonly and consistently reported, most other PROGRESS+characteristics (ie, ethnicity, religion, socioeconomic status, carer health) were either inconsistently or minimally reported.

While a large number of tools were identified, most were only used in a single article, and those used two times were often applied by the same authors. This is in contrast with the relatively high uptake of the CSNAT-I, suggesting a clear preference within both research and practice communities. The widespread adoption of CSNAT-I may reflect a combination of factors, including its theoretical and conceptual grounding, evidence base and the availability of implementation support. We also acknowledge that patterns of tool uptake may be shaped by the influence of established research networks and the availability of funding to support implementation, validation and dissemination activities, which may affect the visibility, dissemination and use of particular tools. This highlights how social and structural dynamics within research and practice communities may also influence which tools are adopted and evaluated, with potential implications for equity in tool development and the diversity of approaches represented in the literature. The dominance of a very small number of tools may additionally influence how caregiver needs are conceptualised and operationalised in practice and research by narrowing the scope of needs assessments. Equity considerations in carer needs assessment involve recognising that carer needs likely differ across socioeconomic, cultural, relational and care contexts and are compounded by structural barriers. Tools need to account for these differences in order to effectively elicit diverse needs and enable the conditions necessary to equitably address needs.

The remaining tools varied in their conceptual focus and intended purpose. Some tools elicited carers’ perceived support needs across multiple domains (eg, information needs or support needs, assessed by the FIN), while others functioned primarily as a screening tool (the CAT). Some tools focused on specific aspects of need, such as spiritual support (eg, desire for prayer, assessed by the SNI). Fourteen articles were excluded as they used tools that measured related but adjacent constructs to need (eg, burden, strain, preparedness, psychological distress). While we applied strict inclusion criteria to identify tools measuring actionable need, the variation in tools suggests the absence of a clear conceptual consensus regarding what constitutes a caregiver support need. This heterogeneity may contribute to inconsistency in how caregiver needs are identified and addressed across research and practice.

Aside from work with the CSNAT-I, there was little evidence of tool evaluation beyond psychometric validation, and the least common evaluation type was adoption or the extent to which tools are implemented within different populations and contexts. Successful uptake of tools across caregiver settings depends not only on psychometric validity but also on their acceptability to caregivers and health practitioners, feasibility within existing service protocols and workflows and alignment with organisational priorities and resources. As such, tools to identify and respond to carer needs that require additional time and documentation are less likely to be adopted unless they are accompanied by sufficient support, including staff training, endorsement by leadership and integration into established service and care processes. Given the substantial and persistent ethical, relational and systemic challenges experienced by health practitioners and caregivers of people at end-of-life and the dominant focus on patient needs,^20 32–34^ the implementation of tools to identify and respond specifically to caregiver needs is likely to be particularly challenging. Therefore, the development of such tools should consider explicit focus on implementation models and frameworks (eg, Consolidated Framework for Implementation Research 2.0,^35^ the integrated Promoting Action on Research Implementation in Health Services framework^36^ or Normalisation Process Theory^37^) that would emphasise greater consideration of individual and organisational contextual factors that influence the successful uptake of such tools into practice. Without due consideration of these factors, caregiver needs assessments will almost certainly not become embedded components of routine care.

While numerous tools exist, their development and evaluation appear to have occurred largely within socially and geographically narrow carer populations (eg, female, spousal and cancer-focused caregiving samples), raising questions around their equitable development and wider applicability to other carer populations. Non-malignant conditions (eg, motor neuron disease, dementia, cardiovascular) involve different trajectories to cancer for patients and their caregivers.^38^ Representation from middle- and low-income countries and of minoritised ethnic groups within high-income countries was minimal, raising questions around whether existing tools sufficiently capture culturally diverse understandings of caregiving. Few articles reported deliberate inclusion of socially marginalised groups in tool development and use, and there was little explicit evidence of cultural and linguistic adaptation of tools used in different settings. Findings suggest that considerations of equity have not yet been central to the development and evaluation of tools to assess and address carer support needs.

Although increasingly recognised as important in palliative care research,^13^ intersectionality theory has not yet been meaningfully operationalised in the development and evaluation of carer support needs assessment tools. Future tool development work could consider how multiple, overlapping social identities and positions (eg, gender, ethnicity, socioeconomic status and caregiving context) interact to shape carers’ experiences and support needs. This might involve purposive inclusion of diverse carer groups in tool development, analysis of how needs differ across intersecting characteristics, and design of tools that are flexible or adaptable to different social and cultural contexts. This approach to tool development could support more equitable and context-sensitive identification and response to carer needs.

### Strengths and limitations

This review has several strengths. It is the first review to examine equity in the development of tools to assess carer support needs. The protocol was preregistered,^22^ and the review was conducted and reported according to recommended guidelines.^23 24^ This review aimed to map the breadth of available tools; searches were performed in a wide range of databases and then updated to identify the latest literature. All stages of screening, data extraction and collation were undertaken by two or more reviewers to ensure accuracy, reduce selection bias, support resolution of disagreements and enhance the overall quality of the review findings.

This review also has limitations. We did not search for grey literature. Articles written in a language other than English were excluded during searches; other relevant tools may have been missed, and articles published in other languages may have changed the overall pattern of PROGRESS+results. We did not undertake a comprehensive review of the psychometric properties of identified tools (eg, using Consensus-based Standards for the selection of health Measurement Instruments guidelines).^39^ It was beyond the scope of this review to consider equity across the identified tools’ lifecycles (ie, equity in tool design, development, application and evaluation), meaning that it is possible such considerations may be present but not explicitly reported. We included some tools which were not specifically developed for use in palliative or end-of-life care but which were used to inform support needs in these settings. It is possible that such tools may reflect broader or more general caregiving experiences rather than needs specific to end-of-life care contexts.

### Implications

This review highlights several implications for future research and practice. First, there is a need for research to advance both equity and intersectionality-informed tool development and validation of caregiver populations, alongside more comprehensive reporting of study samples’ sociodemographic characteristics. Second, due to the existence of multiple instruments, conceptual clarity is needed in the distinction between caregiver needs and related constructs (eg, burden, distress), enabling more direct identification of the support necessary to sustain caregiving. Further work involving formal concept analysis of terms (eg, of caregiver support need) may help to clarify the construct and support more consistent tool development. Third, due to the limited reuse of most tools, research should go beyond psychometric validation to examine the implementation, impact and sustainability of tools used in diverse palliative care settings. For example, this might include studies evaluating how the use of a needs assessment tool influences carer outcomes (eg, unmet need, burden), practitioner decision-making or service provision over time, as well as the feasibility of sustained use within routine care. Fourth, carer assessment tools should be designed with a view to integrating into routine workflows, including clear guidance on tool administration, interpretation, follow-up (ensuring needs assessments are ongoing rather than a one-time activity) and referral pathways to additional services. For example, tools might be embedded within electronic patient record systems with prompts for follow-up conversations or linked to structured care pathways that guide practitioners in responding to identified needs.

## Conclusion

While a large number of tools exist to assess and address carer support needs (with concentrated uptake of a small number of these), there is notable variation in evaluation and the extent to which tools consider equity factors. Existing tools have largely been developed and evaluated within relatively narrow carer populations, with limited and inconsistently reported consideration of equity-related characteristics. There is need for equity-informed tool development and clearer conceptualisation of caregiver needs, alongside efforts to evaluate tool implementation, impact and sustainability in palliative care.

## Supplementary material

10.1136/bmjopen-2026-121897 online supplemental file 1

## Data Availability

Data sharing not applicable as no datasets generated and/or analysed for this study.
