# Supplementary material for: Considerations of equity in the development of tools that identify and respond to end-of-life carer support needs: a scoping review
Source: BMJ Open. 2026 Sep 11;16(9):e121897. doi: 10.1136/bmjopen-2026-121897 (PMC13583838; doi:10.1136/bmjopen-2026-121897)
Supplement: Supplementary file 1 [file bmjopen-2026-121897.reviewer_comments.pdf]

PEER REVIEW HISTORY

BMJ Open publishes all reviews undertaken for accepted manuscripts. Reviewers are asked to complete a checklist review form and are provided with free text boxes to elaborate on their assessment. These free text comments are reproduced below.

ARTICLE DETAILS

Title (Provisional)

Considerations of equity in the development of tools that identify and respond to end-of-life carer support needs: a scoping review

Authors

Long, Hannah; Rowland, Christine; Higgerson, James; Hall, Alex; Urwin, Sean; Brooks, Joanna

---

VERSION 1 - REVIEW

Reviewer **1**

Name **White, Nicola**

Affiliation **University College London, Marie Curie Palliative Care Research Department**

Date **22-May-2026**

COI **None**

Thank you for sending the manuscript titled "Considerations of equity in the development of tools that identify and respond to end-of life carer support needs: a scoping review" for review.

This is a scoping review of tools that identify carer support needs. Overall, it is very well constructed and highlights an important topic, equity in carer needs assessment tools within palliative care, providing evidence in a current cap. The methods are well reported, transparent, and robust. The application of the PROGRESS+ framework is well executed and strengthens the comparability. The authors have been clear about the limitations of the work.

There are some minor suggestions that I think would improve the manuscript overall:

The distinction between a needs assessment tool and adjacent constructs (burden, distress, preparedness) is noted but not fully resolved. The authors could provide more detail in why the Preparedness for Caregiving Scale and the Caregiver Strain Index passed inclusion criteria despite the conceptual distinction, which they draw themselves in the Discussion (p.13). A short clarifying sentence in the Methods would help.

Table 3 presents "reported N (%)" as a binary variable, but the Discussion acknowledges that what was reported often lacked granularity (e.g., occupation broadly defined as employed/retired). The table risks overstating data quality by recording presence of reporting without capturing adequacy of reporting. A note in the table legend, or an additional column flagging quality of reporting, would strengthen this.

The Discussion attributes CSNAT-I dominance partly to "stronger theoretical grounding and robust psychometric properties," but this is asserted rather than evidenced. It may equally reflect the involvement of a well-networked research group (several co-authors are CSNAT-I developers, as declared in competing interests). The authors might briefly acknowledge this sociological dimension, which also has equity implications. The tool uptake may reflect researcher networks rather than inherent tool quality.

Related to the point above, the competing interests statement (CR and AH are co-authors on included CSNAT-I articles) is appropriately disclosed. However, it would be worth explicitly stating in the Methods that these reviewers were excluded not just from screening/extraction of those specific papers, but from any discussions in which CSNAT-I data were interpreted.

The four implications in the Discussion are useful but somewhat generic. Implication 3 (go beyond psychometric validation) and implication 4 (design for workflow integration) would benefit from a concrete example each of what this might look like in practice.

Given that the Introduction references intersectionality theory (reference 11), it is notable that this thread is not picked up in the Discussion or implications. Even a brief paragraph on how future tool development might operationalise intersectional approaches would strengthen the overall paper.

Reference 12 in the main text (Torensma et al.) is cited for intersectionality but appears to be about responsiveness to migrants. The more directly relevant intersectionality citation is Butler et al. (reference 11).

**Reviewer**	**2**
**Name**	**Howard, Michelle**
**Affiliation**	**McMaster University, Dept of Family Medicine**
**Date**	**29-May-2026**
**COI**	**None**

Thank you for the opportunity to review this well executed and clearly written scoping review. The topic is important and the results are credible. I hope the following specific suggestions help enhance the clarity of the manuscript.

Minor – Abstract objectives refers to ‘caregivers’ rather than ‘carers’.

Minor - Pg 10 line 22 -spell out SNI first time used

Results charting – An element of research question 1 is to describe the intended purpose of the tools; however, the approach to extracting and charting these data is not fully clear. The results distinguish between “screening tools” and other types, and between tools addressing single versus multiple domains of need, but it is not evident how these categories were developed. Greater clarity on the process used to generate the categories for tool content and intended purpose—and a brief summary statement synthesizing these findings in the results—would strengthen this section. In addition, the level of detail provided on each tool’s intended purpose is somewhat inconsistent across descriptions (e.g., the FIN tool does not specify how it is intended to be used).

Discussion pg 14 line 26 – ‘Non-malignant conditions….’ - consider adding a reference for this statement.

Discussion pg 14 line 38-40 – You suggest that equity has not appear to have been a central consideration in tool development and evaluation (at least to date), but also note that the review could not assess the full lifecycle of the tools. As written, these points seem inconsistent.

Conclusion – I was surprised to see the claim of variation in tool uptake, as this does not appear to have been assessed in the review—unless the authors are referring simply to the smaller number of papers describing implementation compared with development and evaluation. I am also unsure that “notable variation” accurately captures the findings regarding equity considerations; “variation” implies that some studies addressed these factors while others did not. The results instead suggest that, to date, existing tools have largely been developed with a narrow subset of carers.

Reviewer **3**

Name **Krutter, Simon**

Affiliation **Paracelsus Medical Private University, Institute of Nursing Science and Practice**

Date **17-Jun-2026**

COI **None**

Reviewer’s report

Manuscript ID: bmjopen-2026-121897

Date: 17.06.2026

Title: Considerations of equity in the development of tools that identify and respond to end-of-life carer support needs: a scoping review

Thank you for the opportunity to review the manuscript. The scoping review on equity in the development of tools to identify support needs among caregivers in palliative and end-of-life care addresses an important and under-researched topic. The review presents a clearly defined research gap, draws on a comprehensive search across multiple databases, applies the well known PROGRESS+ framework, and provides clear implications. I have only minor comments as noted below.

Introduction:

Page 4 Line 26: Please don't just focus on the burdens and challenges faced by informal caregivers. Caring for a family member can also be a meaningful and fulfilling experience. This positive aspects could also be acknowledged.

Page 4 Line 44: As there is no single “typical” family caregiver and caregivers are a highly heterogeneous group, the diversity of this group could be pointed out more clearly.

Page 5 Line 16: It is stated that “less is known about whether carer needs assessment tools account for socially stratifying factors in their development, use, or evaluation”. It would be useful to clarify whether this gap is specific to palliative care or reflects a broader blind spot in the development of assessment tools across healthcare fields. Are there any comparable study results from other fields?

Design:

Page 6 Line 36: Please describe what the abbreviation “k” stands for.

Page 7 Line 48: Please also briefly explain what the “+” in PROGRESS+ refers to, as readers may not be familiar with the additional factors captured by this component of the framework.

Page 7 Line 55: I wonder why the PROGRESS+ criteria were also applied to patients? As the research question focuses on caregivers, and patient characteristics do not appear to be part of the inclusion criteria. The rationale to apply PROGRESS+ factors on patients could therefore be clarified.

Discussion:

Page 12 Line 23: It would be helpful to discuss whether the inclusion of studies using instruments that assess more general caregiver needs and were not specifically developed for palliative or end-of-life care (e.g., the Preparedness for Caregiving Scale), may have influenced the findings.

Page 13 Line 26: The finding, that the variation in tools suggests a lack of conceptual consensus regarding what constitutes a caregiver support need, is particularly interesting. Could a concept analysis be recommended for conceptual clarification of caregiver support needs in palliative care to inform future instrument development?

VERSION 1 - AUTHOR RESPONSE

Reviewer 1	The distinction between a needs assessment tool and adjacent constructs (burden, distress, preparedness) is noted but not fully resolved. The authors could provide more detail in why the Preparedness for Caregiving Scale and the Caregiver Strain Index passed inclusion criteria despite the conceptual distinction, which they draw themselves in the Discussion (p.13). A short clarifying sentence in the Methods would help.	Now added (p.7):<br>"While we distinguished between caregiver support needs and other constructs (such as burden, strain, preparedness) and excluded studies using tools that measured these other constructs alone, we included measures where studies explicitly used them to identify or respond to carers' support needs, or where items were interpreted as indicating actionable areas for support."
Reviewer 1	Table 3 presents "reported N (%)" as a binary variable, but the Discussion acknowledges that what was reported often lacked granularity (e.g., occupation broadly defined as employed/retired). The table risks overstating data quality by recording presence of reporting without capturing adequacy of reporting. A note in the table legend, or an additional column flagging quality of reporting, would strengthen this.	Have now amended (table 3) by adding following table legend as suggested:<br><br>"** Reported N (%) reflects whether a characteristic was reported in each study, but does not capture the level, consistency, or granularity of reporting (e.g., broad categories such as "employed/retired" versus more detailed classifications)."
Reviewer 1	The Discussion attributes CSNAT-I dominance partly to "stronger theoretical grounding and robust psychometric properties," but this is asserted rather than evidenced. It may equally reflect the involvement of a well-networked research group (several co-authors are CSNAT-I developers, as declared in competing interests). The authors might briefly acknowledge this sociological dimension, which also has equity implications. The tool uptake may reflect researcher networks rather than inherent tool quality.	Added p.14:<br>"The widespread adoption of CSNAT-I may reflect a combination of factors, including its theoretical and conceptual grounding, evidence base, and the availability of implementation support. We also acknowledge that patterns of tool uptake may be shaped by the influence of established research networks and the availability of funding to support implementation, validation and dissemination activities, which may affect the visibility, dissemination, and use of particular tools. This highlights how social and structural dynamics within research and practice communities may also

		influence which tools are adopted and evaluated, with potential implications for equity in tool development and the diversity of approaches represented in the literature"
Reviewer 1	Related to the point above, the competing interests statement (CR and AH are co-authors on included CSNAT-I articles) is appropriately disclosed. However, it would be worth explicitly stating in the Methods that these reviewers were excluded not just from screening/extraction of those specific papers, but from any discussions in which CSNAT-I data were interpreted.	Added p.7:<br>"Reviewers were not involved in the screening or data extraction of any studies to which they had contributed. Later interpretation of findings was conducted through discussion within the full research team, ensuring transparency, critical input from multiple researchers, and minimising the influence of any individual perspective."
Reviewer 1	The four implications in the Discussion are useful but somewhat generic. Implication 3 (go beyond psychometric validation) and implication 4 (design for workflow integration) would benefit from a concrete example each of what this might look like in practice.	Added p.17-18:<br><br>Re Implication 3: "For example, this might include studies evaluating how the use of a needs assessment tool influences carer outcomes (e.g., unmet need, burden), practitioner decision-making, or service provision over time, as well as the feasibility of sustained use within routine care."<br><br>Re. Implication 4: "For example, tools might be embedded within electronic patient record systems with prompts for follow-up conversations or linked to structured care pathways that guide practitioners in responding to identified needs."
Reviewer 1	Given that the Introduction references intersectionality theory (reference 11), it is notable that this thread is not picked up in the Discussion or implications. Even a brief paragraph on how future tool development might operationalise intersectional approaches would strengthen the overall paper	Reference [11] is now [13]<br><br>Added p. 16:<br>"Although increasingly recognised as important in palliative care research [13], intersectionality theory has not yet been meaningfully operationalised in the development and evaluation of

		carer support needs assessment tools. Future tool development work could consider how multiple, overlapping social identities and positions (e.g., gender, ethnicity, socioeconomic status, and caregiving context) interact to shape carers' experiences and support needs. This might involve purposive inclusion of diverse carer groups in tool development, analysis of how needs differ across intersecting characteristics, and design of tools that are flexible or adaptable to different social and cultural contexts. This approach to tool development could support more equitable and context-sensitive identification and response to carer needs."
Reviewer 1	Reference 12 in the main text (Torensma et al.) is cited for intersectionality but appears to be about responsiveness to migrants. The more directly relevant intersectionality citation is Butler et al. (reference 11).	Amended p.5
Reviewer 2	Minor – Abstract objectives refers to 'caregivers' rather than 'carers'.	Amended p. 2
Reviewer 2	Minor - Pg 10 line 22 -spell out SNI first time used	Amended p.10
Reviewer 2	Results charting – An element of research question 1 is to describe the intended purpose of the tools; however, the approach to extracting and charting these data is not fully clear. The results distinguish between "screening tools" and other types, and between tools addressing single versus multiple domains of need, but it is not evident how these	Now added the following:<br><br>p. 8-9: "Data on tool characteristics and intended purpose were extracted from each article based on descriptions provided by study authors. Categories relating to tool purpose and content were developed iteratively during the data extraction process

	categories were developed. Greater clarity on the process used to generate the categories for tool content and intended purpose—and a brief summary statement synthesizing these findings in the results—would strengthen this section. In addition, the level of detail provided on each tool's intended purpose is somewhat inconsistent across descriptions (e.g., the FIN tool does not specify how it is intended to be used).	through discussion within the research team. Categorisation was guided by how tools were described in the original articles; for example, where authors explicitly identified a tool as a screening instrument, it was classified as such. Tools comprising multiple domains were categorised as multi-domain, while those producing a single overall score were categorised as unidimensional. In cases where domain structure was not explicitly reported, categorisation was based on descriptions of the tool's structure and content."<br><br>p.10-11: "The identified tools predominantly focused on identifying caregiver needs, with a smaller number designed primarily for evaluation or outcome measurement. There was substantial variation in scope, ranging from tools focused on a single domain (e.g., spiritual needs) to multi-domain instruments intended to support broader, more holistic assessment of caregiver support needs."<br><br>p.12 "The tool is intended to assess family caregiving needs in the context of advanced cancer, using ratings of importance and need fulfilment to identify unmet needs that may require support or intervention."
Reviewer 2	Discussion pg 14 line 26 – 'Non-malignant conditions….' - consider adding a reference for this statement.	Added Karagkounis C, et al (2026) [reference 39]- pages 15 and references
Reviewer 2	Discussion pg 14 line 38-40 – You suggest that equity has not appear to have been a central consideration in tool development and evaluation (at least to date), but also note that the review could not assess the full lifecycle of the	Amended so now reads p.17: "It was beyond the scope of this review to consider equity across the identified tools' lifecycles (i.e., equity in tool design, development, application, and evaluation)

	tools. As written, these points seem inconsistent.	meaning that it is possible such considerations may be present but not explicitly reported.”
Reviewer 2	Conclusion – I was surprised to see the claim of variation in tool uptake, as this does not appear to have been assessed in the review—unless the authors are referring simply to the smaller number of papers describing implementation compared with development and evaluation. I am also unsure that “notable variation” accurately captures the findings regarding equity considerations; “variation” implies that some studies addressed these factors while others did not. The results instead suggest that, to date, existing tools have largely been developed with a narrow subset of carers	Now amended page 18 so reads as follows: “While a large number of tools exist to assess and address carer support needs (with concentrated uptake of a small number of these), there is notable variation in evaluation, and the extent to which tools consider equity factors. Existing tools have largely been developed and evaluated within relatively narrow carer populations, with limited and inconsistently reported consideration of equity-related characteristics. There is need for equity-informed tool development and clearer conceptualisation of caregiver needs, alongside efforts to evaluate tool implementation, impact, and sustainability in palliative care.”
Reviewer 3	Page 4 Line 26: Please don't just focus on the burdens and challenges faced by informal caregivers. Caring for a family member can also be a meaningful and fulfilling experience. This positive aspects could also be acknowledged.	Amended/ added so now reads p.4: “Although caregiving can be experienced as meaningful and fulfilling, many carers face high physical, practical, and emotional demands”. We have additionally added reference [8] to support this point.
Reviewer 3	Page 4 Line 44: As there is no single “typical” family caregiver and caregivers are a highly heterogeneous group, the diversity of this group could be pointed out more clearly.	Added p. 4: “Caregivers represent a highly heterogeneous group, varying across characteristics such as age, gender, relationship to the care recipient, cultural and socioeconomic background, health status, and caregiving context, all of which may shape both caregiving experiences and support needs.”
Reviewer 3	Page 5 Line 16: It is stated that “less is known about whether carer needs assessment tools account for socially stratifying factors in their development, use, or	This is an interesting comment and (from what we can find) true of other disciplines too - although we found less explicit recognition of it in the literature

	evaluation". It would be useful to clarify whether this gap is specific to palliative care or reflects a broader blind spot in the development of assessment tools across healthcare fields. Are there any comparable study results from other field	than we expected. Added p. 5: "While some evidence suggests that certain groups of caregivers experience distinct or inadequately met needs [15], even less is known about whether carer needs assessment tools account for socially stratifying factors in their development, use, or evaluation. This reflects a wider methodological gap, not only in the carer literature [11], but in clinical and health outcome measurement across research fields (e.g., cancer [17], allergy and immunology[18], rheumatology [19]), where tools have often been developed and validated without explicit attention to equity."
Reviewer 3	Page 6 Line 36: Please describe what the abbreviation "k" stands for.	Added p. 8: "A second reviewer extracted data in stage 1 for 20% (k=25; where 'k' denotes the number of articles) of the eligible articles."
Reviewer 3	Page 7 Line 48: Please also briefly explain what the "+" in PROGRESS+ refers to, as readers may not be familiar with the additional factors captured by this component of the framework.	Added/ amended p. 8: "In stage 2, we used PROGRESS+ [16] – an acronym referring to socially-stratifying details (e.g., place of residence; race/ethnicity/culture/language; occupation; gender/sex; religion; education; socioeconomic status; social capital plus other context specific factors such as age, health condition, features of relationship) - to identify and extract sample characteristics associated with equity in studies with carer participants"
Reviewer 3	Page 7 Line 55: I wonder why the PROGRESS+ criteria were also applied to patients? As the research question focuses on caregivers, and patient characteristics do not appear to be	Added p. 8: "This reflects the relational nature of caregiving in palliative and end-of-life care, where patient characteristics (e.g., diagnosis, disease trajectory, and

	part of the inclusion criteria. The rationale to apply PROGRESS+ factors on patients could therefore be clarified.	sociodemographic context) shape caregiving roles, experiences, and associated support needs.”
Reviewer 3	Page 12 Line 23: It would be helpful to discuss whether the inclusion of studies using instruments that assess more general caregiver needs and were not specifically developed for palliative or end-of-life care (e.g., the Preparedness for Caregiving Scale), may have influenced the findings.	Added p.17:<br>“We included some tools which were not specifically developed for use in palliative or end-of-life care but which were used to inform support needs in these settings. It is possible that such tools may reflect broader or more general caregiving experiences rather than needs specific to end-of-life care contexts.”
Reviewer 3	Page 13 Line 26: The finding, that the variation in tools suggests a lack of conceptual consensus regarding what constitutes a caregiver support need, is particularly interesting. Could a concept analysis be recommended for conceptual clarification of caregiver support needs in palliative care to inform future instrument development?	Added p.17:<br>“Further work involving formal concept analysis of terms (e.g. of caregiver support need) may help to clarify the construct and support more consistent tool development.”

VERSION 2 - REVIEW

Reviewer **1**

Name **White, Nicola**

Affiliation **University College London, Marie Curie Palliative Care Research Department**

Date **17-Jul-2026**

COI

Thank you. The authors have responded to all comments and amended the manuscript appropriately. The manuscript now is very clear and transparent in the reporting. Thank you.

Reviewer **2**

Name **Howard, Michelle**

Affiliation McMaster University, Dept of Family Medicine

Date 30-Jul-2026

COI

No additional suggestions, all comments have been addressed.
