# Supplementary material for: Considerations of equity in the development of tools that identify and respond to end-of-life carer support needs: a scoping review
Source: BMJ Open. 2026 Sep 11;16(9):e121897. doi: 10.1136/bmjopen-2026-121897 (PMC13583838; doi:10.1136/bmjopen-2026-121897)
Supplement: Supplementary file 2 [file bmjopen-16-9-s001.docx]

**Supplementary Materials 1 – Published scoping review protocol**

**Supplementary Materials 2 – PRISMA flow diagram and full article references**

Records identified through database searching
(n=516)

## Identification

Duplicates removed (n=160)

Records after duplicates removed
(n=356)

## Screening

Records screened by title and abstract
(n=356)

Records excluded (n=255)

Full text articles assessed

(n=101)

Full text articles excluded (with reasons) (n=39)

- Does not use tool to assess carer support needs / No eligible support needs tool n=13
- Wrong publication type (background/commentary/not a study report) n=4
- Not end of life care n=6
- Systematic review n=3
- Wrong population n=8
- Wrong outcome n=2
- Duplicate n=1
- No full text available n=1
- Non-English language n=1

## Eligibility

Eligible articles (n=62)

Articles identified via backward and forward searching (n=27)

## Included

Full text articles excluded (with reasons) (n=15)

- Wrong population n=11
- Wrong outcome n=3
- Does not use tool n=1

Total articles included in scoping review (n=74)

Carer population articles (n=64)

1. Alvariza, A., Holm, M., Benkel, I., Norinder, M., Ewing, G., Grande, G., Håkanson, C., Öhlen, J., Årestedt, K. A person-centred approach in nursing: Validity and reliability of the Carer Support Needs Assessment Tool. European Journal of Oncology Nursing 2018; 35: 1-8. https://doi.org/10.1016/j.ejon.2018.04.005.
2. Aoun S, Deas K, Toye C, Ewing G, Grande G, Stajduhar K. Supporting family caregivers to identify their own needs in end-of-life care: Qualitative findings from a stepped wedge cluster trial. Palliative Medicine. 2015;29(6):508-517. doi:10.1177/0269216314566061
3. Aoun SM, Deas K, Howting D, Lee G (2015c) Exploring the Support Needs of Family Caregivers of Patients with Brain Cancer Using the CSNAT: A Comparative Study with Other Cancer Groups. PLoS ONE 10(12): e0145106. https://doi.org/10.1371/journal.pone.0145106
4. Aoun SM, Grande G, Howting D, Deas K, Toye C, Troeung L, et al. (2015d) The Impact of the Carer Support Needs Assessment Tool (CSNAT) in Community Palliative Care Using a Stepped Wedge Cluster Trial. PLoS ONE 10(4): e0123012. https://doi.org/10.1371/journal.pone.0123012
5. Aoun SM, Deas K, Kristjanson LJ, Kissane DW. Identifying and addressing the support needs of family caregivers of people with motor neurone disease using the Carer Support Needs Assessment Tool. Palliat Support Care. 2017 Feb;15(1):32-43. doi: 10.1017/S1478951516000341.
6. Aoun SM, Toye C, Slatyer S, Robinson A, Beattie E. A person-centred approach to family carer needs assessment and support in dementia community care in Western Australia. Health Soc Care Community. 2018 Jul;26(4):e578-e586. doi: 10.1111/hsc.12575.
7. Aoun SM, O'Brien MR, Knighting K. Using the Carers' Alert Thermometer tool to identify needs and support family caregivers of people with motor neurone disease: moving beyond needs assessments. Palliat Care Soc Pract. 2024 Feb 11;18:26323524241228306. doi: 10.1177/26323524241228306.
8. Bijnsdorp FM, Pasman HRW, Boot CRL, van Hooft SM, van Staa A, Francke AL. Profiles of family caregivers of patients at the end of life at home: a Q-methodological study into family caregiver' support needs. BMC Palliat Care. 2020 Apr 21;19(1):51. doi: 10.1186/s12904-020-00560-x.
9. Brazil K, Kaasalainen S, Williams A, Dumont S. A comparison of support needs between rural and urban family caregivers providing palliative care. Am J Hosp Palliat Care. 2014 Feb;31(1):13-9. doi: 10.1177/1049909112474712.
10. Buck HG, McMillan SC. A psychometric analysis of the spiritual needs inventory in informal caregivers of patients with cancer in hospice home care. Oncol Nurs Forum. 2012 Jul;39(4):E332-9. doi: 10.1188/12.ONF.E332-E339.
11. Bužgová R, Špatenková N, Fukasová-Hajnová E, Feltl D. Assessing needs of family members of inpatients with advanced cancer. Eur J Cancer Care (Engl). 2016 Jul;25(4):592-9. doi: 10.1111/ecc.12441.
12. Carduff E, Jarvis A, Highet G, Finucane A, Kendall M, Harrison N, Greenacre J, Murray SA. Piloting a new approach in primary care to identify, assess and support carers of people with terminal illnesses: a feasibility study. BMC Fam Pract. 2016 Feb 11;17:18. doi: 10.1186/s12875-016-0414-2.
13. Chan WCH. Future provision of home end-of-life care: Family carers’ willingness for caregiving and needs for support. Palliative and Supportive Care 2021: 19, 580–586. https://doi.org/10.1017/S1478951520001273
14. Chan WCH, Yu CTK,Kwok DKS, Wan JKM. Prevalence of and factors associated with demoralization among family caregivers of palliative care patients in Hong Kong. Palliative and Supportive Care 2023. https://doi.org/10.1017/S1478951523000950
15. Cheng HL, Leung DYP, Ko PS, Chung MW, Lam WM, Lam PT, Luk AL, Lam SC. Reliability, validity and acceptability of the traditional Chinese version of the carer support needs assessment tool in Hong Kong palliative care settings. BMC Palliat Care. 2021 Oct 9;20(1):152. doi: 10.1186/s12904-021-00852-w.
16. Cheng HL, Leung DYP, Ko PS, Lam WM, Lam PT, Luk AL, Chung MW, Lam SC. Mediating role of self-efficacy between unmet needs and quality of life in palliative cancer caregivers. Psychooncology. 2023 Mar;32(3):457-464. doi: 10.1002/pon.6099.
17. Du Preez J, Celenza A, Etherton-Beer C, Moffat P, Campbell E, Arendts G. Caring for persons with Dementia: a qualitative study of the needs of carers following care recipient discharge from hospital. BMC Palliat Care. 2023 Dec 13;22(1):200. doi: 10.1186/s12904-023-01322-1.
18. DuBenske LL, Wen KY, Gustafson DH, Guarnaccia CA, Cleary JF, Dinauer SK, McTavish FM. Caregivers' differing needs across key experiences of the advanced cancer disease trajectory. Palliat Support Care. 2008 Sep;6(3):265-72. doi: 10.1017/S1478951508000400.
19. Durepos, P., Akhtar-Danesh, N., Sussman, T., Ploeg, J., Boerner, K., Kaasalainen, S. Evaluation of the Caring Ahead: Preparing for End-of-Life With Dementia Questionnaire, Journal of the American Medical Directors Association. 2021; 22(10), 2108-2114.e4, https://doi.org/10.1016/j.jamda.2021.06.032.
20. Ewing G, Brundle C, Payne S, Grande G; National Association for Hospice at Home. The Carer Support Needs Assessment Tool (CSNAT) for use in palliative and end-of-life care at home: a validation study. J Pain Symptom Manage. 2013 Sep;46(3):395-405. doi: 10.1016/j.jpainsymman.2012.09.008.
21. Ewing G, Croke S, Rowland C, et al. Suitability and acceptability of the Carer Support Needs Assessment Tool (CSNAT) for the assessment of carers of people with MND: a qualitative study. BMJ Open 2020;10:e039031. doi:10.1136/bmjopen-2020-039031
22. Greene A, Aranda S, Tieman JJ, Fazekas B, Currow DC. Can assessing caregiver needs and activating community networks improve caregiver-defined outcomes? A single-blind, quasi-experimental pilot study: community facilitator pilot. Palliat Med. 2012 Oct;26(7):917-23. doi: 10.1177/0269216311421834.
23. Johnson MJ, Jamali A, Ross J, Fairhurst C, Boland J, Reigada C, Hart SP, Grande G, Currow DC, Wells AU, Bajwah S, Papadopoulos T, Bland JM, Yorke J. Psychometric validation of the needs assessment tool: progressive disease in interstitial lung disease. Thorax. 2018 Sep;73(9):880-883. doi: 10.1136/thoraxjnl-2017-210911.
24. Johnston B, Patterson A, Bird L, Wilson E, Almack K, Mathews G, Seymour J. Impact of the Macmillan specialist Care at Home service: a mixed methods evaluation across six sites. BMC Palliat Care. 2018 Feb 23;17(1):36. doi: 10.1186/s12904-018-0281-9.
25. King C, Khamis A, Ross J, Murtagh FEM, Johnson MJ, Ramsenthaler C. Concurrent Validity and Prognostic Utility of the Needs Assessment Tool: Progressive Disease Heart Failure. J Pain Symptom Manage. 2022 May;63(5):635-644.e3. doi: 10.1016/j.jpainsymman.2022.01.014.
26. Knighting K, O'Brien MR, Roe B, Gandy R, Lloyd-Williams M, Nolan M, Jack BA. Development of the Carers' Alert Thermometer (CAT) to identify family carers struggling with caring for someone dying at home: a mixed method consensus study. BMC Palliat Care. 2015a May 3;14:22. doi: 10.1186/s12904-015-0010-6.
27. Knighting K, O'Brien MR, Roe B, Gandy R, Lloyd-Williams M, Nolan M, Jack BA. Gaining consensus on family carer needs when caring for someone dying at home to develop the Carers' Alert Thermometer (CAT): a modified Delphi study. J Adv Nurs. 2016 Jan;72(1):227-39. doi: 10.1111/jan.12752.
28. Kreyer C, Bükki J, Pleschberger S. Development of a German version of the Carer Support Needs Assessment Tool (CSNAT): The process of translation and cultural adaptation. Palliat Support Care. 2020 Apr;18(2):193-198. doi: 10.1017/S1478951519000671.
29. Laabar TD, Auret K, Saunders C, Ngo H, Johnson CE. Support Needs for Bhutanese Family Members Taking Care of Loved Ones Diagnosed with Advanced Illness. J Palliat Care. 2022 Jul;37(3):401-409. doi: 10.1177/08258597211066248.
30. Lund L, Ross L, Petersen MA, Rosted E, Bollig G, Juhl GI, Farholt H, Winther H, Laursen L, Blaaberg EG, Weensgaard S, Guldin MB, Ewing G, Grande G, Groenvold M. Process, content, and experiences of delivering the Carer Support Needs Assessment Tool Intervention (CSNAT-I) in the Danish specialised palliative care setting. Support Care Cancer. 2022 Jan;30(1):377-387. doi: 10.1007/s00520-021-06432-1.
31. Malewezi E, O'Brien M, , Knighting K, Thomas J, Jack B. Development of the Carers’ Alert Thermometer for Stroke Family Caregivers (CAT-S): A Multiphase Action Research Study. Health and Social Care in the Community. 2023 Feb 27; 1660169. doi: 10.1155/2023/1660169
32. Marston C, Morgan DD, Philip J, Agar MR. Experience and acceptability of a carer-focussed intervention in acute oncology settings: A qualitative study of people with advanced cancer and their carers. Aust Occup Ther J. 2023 Oct;70(5):570-580. doi: 10.1111/1440-1630.12887.
33. McIlfatrick S, Doherty LC, Murphy M, Dixon L, Donnelly P, McDonald K, Fitzsimons D. 'The importance of planning for the future': Burden and unmet needs of caregivers' in advanced heart failure: A mixed methods study. Palliat Med. 2018 Apr;32(4):881-890. doi: 10.1177/0269216317743958.
34. McMillan SC, Small BJ, Haley WE. Improving hospice outcomes through systematic assessment: a clinical trial. Cancer Nurs. 2011 Mar-Apr;34(2):89-97. doi: 10.1097/NCC.0b013e3181f70aee.
35. Neo SH, Yang GM, Kanesvaran R, Cheung YB. Translation and Validation of the 10-Item FAMCARE Scale to Assess Satisfaction of Family Caregivers With Care Given to Cancer Patients. J Pain Symptom Manage. 2019 Nov;58(5):878-885.e2. doi: 10.1016/j.jpainsymman.2019.07.018.
36. Norinder M, Årestedt K, Lind S, Axelsson L, Grande G, Ewing G, Holm M, Öhlén J, Benkel I, Alvariza A. Higher levels of unmet support needs in spouses are associated with poorer quality of life - a descriptive cross-sectional study in the context of palliative home care. BMC Palliat Care. 2021 Aug 28;20(1):132. doi: 10.1186/s12904-021-00829-9.
37. Norinder M, Årestedt K, Axelsson L, Grande G, Ewing G, Alvariza A. Increased preparedness for caregiving among family caregivers in specialized home care by using the Carer Support Needs Assessment Tool Intervention. Palliat Support Care. 2024 Apr;22(2):236-242. doi: 10.1017/S1478951523000639.
38. Norinder M, Axelsson L, Årestedt K, Grande G, Ewing G, Alvariza A. Family caregivers' experiences of discussing their needs with a nurse during specialised home care utilizing the carer support needs assessment tool intervention - A qualitative study. Eur J Oncol Nurs. 2023 Oct;66:102412. doi: 10.1016/j.ejon.2023.102412.
39. Osse BH, Vernooij-Dassen MJ, Schadé E, Grol RP. Problems experienced by the informal caregivers of cancer patients and their needs for support. Cancer Nurs. 2006 Sep-Oct;29(5):378-88; quiz 389-90. doi: 10.1097/00002820-200609000-00005.
40. Papadakos J, Agarwal A, Charow R, Quartey NK, D'souza A, Giuliani M, Millar BA, Massey C, Shultz D, Chung C. Informational needs of brain metastases patients and their caregivers. Neurooncol Pract. 2019 Jan;6(1):47-60. doi: 10.1093/nop/npy008.
41. Pawlow PC, Blumenthal NP, Christie JD, Matura LA, Aryal S, Ersek M. The Supportive Care Needs of Primary Caregivers of Lung Transplant Candidates. J Pain Symptom Manage. 2021 Nov;62(5):918-926. doi: 10.1016/j.jpainsymman.2021.05.004.
42. Pidgeon TM, Johnson CE, Lester L, Currow D, Yates P, Allingham SF, Bird S, Eagar K. Perceptions of the care received from Australian palliative care services: A caregiver perspective. Palliat Support Care. 2018 Apr;16(2):198-208. doi: 10.1017/S1478951517000177.
43. Purba CIH, Johnston B, Kotronoulas G. An Exploration of Family Caregivers' Health Care Needs When Caring for Patients With Cancer in the Resource-Challenged Context of West Java, Indonesia. Semin Oncol Nurs. 2023 Jun;39(3):151369. doi: 10.1016/j.soncn.2022.151369.
44. Scott G, Whyler N, Grant G. A study of family carers of people with a life-threatening illness 1: the carers' needs analysis. Int J Palliat Nurs. 2001 Jun;7(6):290-1. doi: 10.12968/ijpn.2001.7.6.9027.
45. Steele RG, Fitch MI. Needs of family caregivers of patients receiving home hospice care for cancer. Oncol Nurs Forum. 1996 Jun;23(5):823-8. PMID: 8792351.
46. Thomsen KT, Guldin MB, Nielsen MK, Ollars CL, Jensen AB. A process evaluation of systematic risk and needs assessment for caregivers in specialised palliative care. BMC Palliat Care. 2017 Apr 8;16(1):23. doi: 10.1186/s12904-017-0196-x.
47. Zapata CB, Dionne-Odom JN, Harris HA, Kalanithi L, Lin J, Bischoff K, Fazzalaro K, Pantilat SZ. Honoring What We Say We Do: Developing Real-World Tools for Routine Family Caregiver Assessment and Support in Outpatient Palliative Care. J Palliat Med. 2023 Mar;26(3):376-384. doi: 10.1089/jpm.2022.0043.
48. Zhou S, Zhao Q, Weng H, Wang N, Wu X, Li X, Zhang L. Translation, cultural adaptation and validation of the Chinese version of the Carer Support Needs Assessment Tool for family caregivers of cancer patients receiving home-based hospice care. BMC Palliat Care. 2021 May 19;20(1):71. doi: 10.1186/s12904-021-00766-7.
49. Becqué YN, Rietjens JAC, van der Heide A, Witkamp E. Failed implementation of a nursing intervention to support family caregivers: An evaluation study using Normalization Process Theory. J Adv Nurs. 2025 Feb;81(2):937-952. doi: 10.1111/jan.16261.
50. Buck, H., McMillan, S.C.  The Unmet Spiritual Needs of Caregivers of Patients With Advanced Cancer. Journal of Hospice & Palliative Nursing 2008; 10(2); 91-99. DOI: 10.1097/01.NJH.0000306737.08850.71
51. Bužgová R, Kozáková R. Psychometric evaluation of a Czech version of the Family Inventory of Needs. Palliat Support Care. 2016 Apr;14(2):142-50. doi: 10.1017/S1478951515001029.
52. Fukui S. Information needs and the related variables of Japanese family caregivers of terminally ill cancer patients. Nurs Health Sci. 2004 Mar;6(1):29-36. doi: 10.1111/j.1442-2018.2003.00170.x.
53. Ham L, Fransen HP, de Graeff A, Hendriks MP, de Jong WK, Kloover J, Kuip E, Mandigers C, Sommeijer D, van de Poll L, Raijmakers N, van Zuylen L. Relatives' Unmet Needs in the Last Year of Life of Patients With Advanced Cancer: Results of a Dutch Prospective, Longitudinal Study (eQuiPe). J Palliat Care. 2025 Jan;40(1):41-50. doi: 10.1177/08258597241239614.
54. Huang LT, Tai CY, Longcoy J, McMillan SC. The Mutual Effects of Perceived Spiritual Needs on Quality of Life in Patients With Advanced Cancer and Family Caregivers. J Hosp Palliat Nurs. 2021 Aug 1;23(4):323-330. doi: 10.1097/NJH.0000000000000758.
55. Kreyer C, Stecher B, Pleschberger S, Ewing G. What individual needs do family caregivers have in palliative home care and how are they supported? A qualitative study of a supportive intervention. Support Care Cancer. 2024 Oct 18;32(11):733. doi: 10.1007/s00520-024-08904-6.
56. Kristjanson LJ, Atwood J, Degner LF. Validity and reliability of the family inventory of needs (FIN): measuring the care needs of families of advanced cancer patients. J Nurs Meas. 1995 Winter;3(2):109-26.
57. Lin WC, Tsao CJ. Information needs of family caregivers of terminal cancer patients in Taiwan. Am J Hosp Palliat Care. 2004 Nov-Dec;21(6):438-44. doi: 10.1177/104990910402100609.
58. Ullrich A, Bergelt C, Marx G, Daubmann A, Benze G, Heine J, Dickel LM, Wowretzko F, Zhang Y, Bokemeyer C, Nauck F, Oechsle K. The CAREPAL-8: a short screening tool for multidimensional family caregiver burden in palliative care. BMC Palliat Care. 2024 Aug 2;23(1):195. doi: 10.1186/s12904-024-01480-w.
59. Lund L, Ross L, Petersen MA, Blach A, Rosted E, Bollig G, Juhl GI, Farholt HB, Winther H, Laursen L, Hasse M, Weensgaard S, Guldin MB, Ewing G, Grande G, Groenvold M. Effect of the Carer Support Needs Assessment Tool intervention (CSNAT-I) in the Danish specialised palliative care setting: a stepped-wedge cluster randomised controlled trial. BMJ Support Palliat Care. 2020 Oct 28:bmjspcare-2020-002467. doi: 10.1136/bmjspcare-2020-002467.
60. Sağlam Z, Koç Z. The validity and reliability of the Turkish version of the Family Inventory of Needs. Palliat Support Care. 2022 Apr;20(2):255-263. doi: 10.1017/S1478951521000833.
61. Schur S, Neubauer M, Amering M, Ebert-Vogel A, Masel EK, Sibitz I, Watzke H, Schrank B. Validation of the Family Inventory of Needs (FIN) for family caregivers in palliative care. Palliat Support Care. 2015 Jun;13(3):485-91. doi: 10.1017/S1478951514000261.
62. Ullrich A, Ascherfeld L, Marx G, Bokemeyer C, Bergelt C, Oechsle K. Quality of life, psychological burden, needs, and satisfaction during specialized inpatient palliative care in family caregivers of advanced cancer patients. BMC Palliat Care. 2017 May 10;16(1):31. doi: 10.1186/s12904-017-0206-z.
63. Wang L, Li Y, Zhao R, Li H. Development and psychometric evaluation of the needs assessment questionnaire for family caregivers of end-of-life older adults in home hospice care. Sci Rep. 2025 Apr 16;15(1):13146. doi: 10.1038/s41598-025-97475-5.
64. Fridriksdottir N, Sigurdardottir V, Gunnarsdottir S. Important needs of families in acute and palliative care settings assessed with the family inventory of needs. Palliat Med. 2006 Jun;20(4):425-32. doi: 10.1191/0269216306pm1148oa. PMID: 16875113Friedriksdottir

Non-carer population articles (n=12)

65. Aoun S, Toye C, Deas K, Howting D, Ewing G, Grande G, Stajduhar K. Enabling a family caregiver-led assessment of support needs in home-based palliative care: Potential translation into practice. Palliat Med. 2015b Dec;29(10):929-38. doi: 10.1177/0269216315583436.
66. Austin L, Ewing G, Grande G. Factors influencing practitioner adoption of carer-led assessment in palliative homecare: A qualitative study of the use of the Carer Support Needs Assessment Tool (CSNAT). PLoS One. 2017 Jun 16;12(6):e0179287. doi: 10.1371/journal.pone.0179287.
67. Diffin J, Ewing G, Harvey G, Grande G. Facilitating successful implementation of a person-centred intervention to support family carers within palliative care: a qualitative study of the Carer Support Needs Assessment Tool (CSNAT) intervention. BMC Palliat Care. 2018 Dec 20;17(1):129. doi: 10.1186/s12904-018-0382-5.
68. Diffin J, Ewing G, Harvey G, Grande G. The Influence of Context and Practitioner Attitudes on Implementation of Person-Centered Assessment and Support for Family Carers Within Palliative Care. Worldviews Evid Based Nurs. 2018b Oct;15(5):377-385. doi: 10.1111/wvn.12323.
69. Ewing G, Austin L, Diffin J, Grande G. Developing a person-centred approach to carer assessment and support. Br J Community Nurs. 2015 Dec;20(12):580-4. doi: 10.12968/bjcn.2015.20.12.580.
70. Ewing G, Austin L, Grande G. The role of the Carer Support Needs Assessment Tool in palliative home care: A qualitative study of practitioners' perspectives of its impact and mechanisms of action. Palliat Med. 2016 Apr;30(4):392-400. doi: 10.1177/0269216315596662.
71. Hall A, Ewing G, Rowland C, Grande G. A drive for structure: A longitudinal qualitative study of the implementation of the Carer Support Needs Assessment Tool (CSNAT) intervention during hospital discharge at end of life. Palliat Med. 2020 Sep;34(8):1088-1096. doi: 10.1177/0269216320930935.
72. Horseman Z, Milton L, Finucane A. Barriers and facilitators to implementing the Carer Support Needs Assessment Tool in a community palliative care setting. Br J Community Nurs. 2019 Jun 2;24(6):284-290. doi: 10.12968/bjcn.2019.24.6.284.
73. O’Brien, M., Jack, B., Kinloch, K., Clabburn, O., Knighting, K. The Carers’ Alert Thermometer (CAT): identifying the support needs of family carers of people living with Motor Neurone Disease. British Journal of Neuroscience Nursing, 2019, 15(3).
74. Allgar VL, Chen H, Richfield E, Currow D, Macleod U, Johnson MJ. Psychometric Properties of the Needs Assessment Tool-Progressive Disease Cancer in U.K. Primary Care. J Pain Symptom Manage. 2018 Oct;56(4):602-612. doi: 10.1016/j.jpainsymman.2018.07.002.

**Supplementary Materials 3 – PRISMA-SCR reporting checklist**

**Preferred Reporting Items for Systematic reviews and Meta-Analyses extension for Scoping Reviews (PRISMA-ScR) Checklist**

| **SECTION** | **ITEM** | **PRISMA-ScR CHECKLIST ITEM** | **REPORTED ON PAGE #** |
| --- | --- | --- | --- |
| **TITLE** | | | |
| Title | 1 | Identify the report as a scoping review. | 1 |
| **ABSTRACT** | | | |
| Structured summary | 2 | Provide a structured summary that includes (as applicable): background, objectives, eligibility criteria, sources of evidence, charting methods, results, and conclusions that relate to the review questions and objectives. | 2 |
| **INTRODUCTION** | | | |
| Rationale | 3 | Describe the rationale for the review in the context of what is already known. Explain why the review questions/objectives lend themselves to a scoping review approach. | 4-5 |
| Objectives | 4 | Provide an explicit statement of the questions and objectives being addressed with reference to their key elements (e.g., population or participants, concepts, and context) or other relevant key elements used to conceptualize the review questions and/or objectives. | 5 |
| **METHODS** | | | |
| Protocol and registration | 5 | Indicate whether a review protocol exists; state if and where it can be accessed (e.g., a Web address); and if available, provide registration information, including the registration number. | 5, Supp Matt 1 |
| Eligibility criteria | 6 | Specify characteristics of the sources of evidence used as eligibility criteria (e.g., years considered, language, and publication status), and provide a rationale. | 6, Table 1 |
| Information sources* | 7 | Describe all information sources in the search (e.g., databases with dates of coverage and contact with authors to identify additional sources), as well as the date the most recent search was executed. | 6 |
| Search | 8 | Present the full electronic search strategy for at least 1 database, including any limits used, such that it could be repeated. | Supp Mat 3 |
| Selection of sources of evidence† | 9 | State the process for selecting sources of evidence (i.e., screening and eligibility) included in the scoping review. | 6-7, Table 1 |
| Data charting process‡ | 10 | Describe the methods of charting data from the included sources of evidence (e.g., calibrated forms or forms that have been tested by the team before their use, and whether data charting was done independently or in duplicate) and any processes for obtaining and confirming data from investigators. | 7-8 |
| Data items | 11 | List and define all variables for which data were sought and any assumptions and simplifications made. | n/a |
| Critical appraisal of individual sources of evidence§ | 12 | If done, provide a rationale for conducting a critical appraisal of included sources of evidence; describe the methods used and how this information was used in any data synthesis (if appropriate). | n/a |
| Synthesis of results | 13 | Describe the methods of handling and summarizing the data that were charted. | 7-8 |
| **RESULTS** | | | |
| Selection of sources of evidence | 14 | Give numbers of sources of evidence screened, assessed for eligibility, and included in the review, with reasons for exclusions at each stage, ideally using a flow diagram. | Supp Mat 2 |
| Characteristics of sources of evidence | 15 | For each source of evidence, present characteristics for which data were charted and provide the citations. | Supp Mat 2 |
| Critical appraisal within sources of evidence | 16 | If done, present data on critical appraisal of included sources of evidence (see item 12). | n/a |
| Results of individual sources of evidence | 17 | For each included source of evidence, present the relevant data that were charted that relate to the review questions and objectives. | Available upon request |
| Synthesis of results | 18 | Summarize and/or present the charting results as they relate to the review questions and objectives. | 9-12, Tables 2 and 3. Supp Mat 5 and 6. |
| **DISCUSSION** | | | |
| Summary of evidence | 19 | Summarize the main results (including an overview of concepts, themes, and types of evidence available), link to the review questions and objectives, and consider the relevance to key groups. | 12 |
| Limitations | 20 | Discuss the limitations of the scoping review process. | 3, 14-15 |
| Conclusions | 21 | Provide a general interpretation of the results with respect to the review questions and objectives, as well as potential implications and/or next steps. | 2, 15 |
| **FUNDING** | | | |
| Funding | 22 | Describe sources of funding for the included sources of evidence, as well as sources of funding for the scoping review. Describe the role of the funders of the scoping review. | 18 |

JBI = Joanna Briggs Institute; PRISMA-ScR = Preferred Reporting Items for Systematic reviews and Meta-Analyses extension for Scoping Reviews.

* Where *sources of evidence* (see second footnote) are compiled from, such as bibliographic databases, social media platforms, and Web sites.

† A more inclusive/heterogeneous term used to account for the different types of evidence or data sources (e.g., quantitative and/or qualitative research, expert opinion, and policy documents) that may be eligible in a scoping review as opposed to only studies. This is not to be confused with *information sources* (see first footnote).

‡ The frameworks by Arksey and O’Malley (6) and Levac and colleagues (7) and the JBI guidance (4, 5) refer to the process of data extraction in a scoping review as data charting*.*

§ The process of systematically examining research evidence to assess its validity, results, and relevance before using it to inform a decision. This term is used for items 12 and 19 instead of "risk of bias" (which is more applicable to systematic reviews of interventions) to include and acknowledge the various sources of evidence that may be used in a scoping review (e.g., quantitative and/or qualitative research, expert opinion, and policy document).

*From:* Tricco AC, Lillie E, Zarin W, O'Brien KK, Colquhoun H, Levac D, et al. PRISMA Extension for Scoping Reviews (PRISMAScR): Checklist and Explanation. Ann Intern Med. 2018;169:467–473. [doi: 10.7326/M18-0850](http://annals.org/aim/fullarticle/2700389/prisma-extension-scoping-reviews-prisma-scr-checklist-explanation).

**Supplementary Materials 4 – Search strings for all electronic databases**

**Medline (Ovid)**

| **#** | **Query** | **Results from 21 May 2025** |
| --- | --- | --- |
| 1 | Terminal Care/ | 33,581 |
| 2 | end of life.mp. | 30,542 |
| 3 | end-of-life care.mp. | 13,840 |
| 4 | last year of life.mp. | 835 |
| 5 | palliative.mp. | 101,236 |
| 6 | Palliative Care/ | 67,172 |
| 7 | Palliative Medicine/ | 628 |
| 8 | "Hospice and Palliative Care Nursing"/ | 2,615 |
| 9 | palliative care.mp. | 78,525 |
| 10 | life limiting.mp. | 2,850 |
| 11 | terminal illness.mp. | 2,007 |
| 12 | advanced illness.mp. | 575 |
| 13 | progressive illness.mp. | 266 |
| 14 | life-threatening illness.mp. | 1,743 |
| 15 | 1 or 2 or 3 or 4 or 5 or 6 or 7 or 8 or 9 or 10 or 11 or 12 or 13 or 14 | 134,853 |
| 16 | carer*.mp. | 18,161 |
| 17 | Caregivers/ | 57,325 |
| 18 | caregiv*.mp. | 110,485 |
| 19 | family caregiver.mp. | 2,472 |
| 20 | informal care*.mp. | 7,852 |
| 21 | unpaid care*.mp. | 693 |
| 22 | family care*.mp. | 14,957 |
| 23 | lay care*.mp. | 244 |
| 24 | elder care.mp. | 720 |
| 25 | home care.mp. | 56,448 |
| 26 | home-based care.mp. | 1,160 |
| 27 | 16 or 17 or 18 or 19 or 20 or 21 or 22 or 23 or 24 or 25 or 26 | 172,655 |
| 28 | support need*.mp. | 4,569 |
| 29 | carer* need*.mp. | 378 |
| 30 | supportive care need*.mp. | 1,046 |
| 31 | 28 or 29 or 30 | 5,868 |
| 32 | need* assessment*.mp. | 38,750 |
| 33 | Needs Assessment/ | 33,424 |
| 34 | carer* assessment*.mp. | 93 |
| 35 | instrument.mp. | 127,804 |
| 36 | carer support needs assessment tool.mp. | 50 |
| 37 | CSNAT.mp. | 43 |
| 38 | instrument development.mp. | 1,948 |
| 39 | 32 or 33 or 34 or 35 or 36 or 37 or 38 | 165,727 |
| 40 | 15 and 27 and 31 and 39 | 108 |
| 41 | limit 40 to english language | 106 |
| 42 | limit 41 to yr="2024 -Current" | 13 |

**PsycINFO (Ovid)**

| **#** | **Query** | **Results from 21 May 2025** |
| --- | --- | --- |
| 1 | end of life.mp. | 13,366 |
| 2 | end-of-life care.mp. | 6,515 |
| 3 | last year of life.mp. | 242 |
| 4 | palliative.mp. | 21,806 |
| 5 | palliative care.mp. | 20,367 |
| 6 | exp Palliative Care/ | 19,230 |
| 7 | life limiting.mp. | 1,151 |
| 8 | terminal illness.mp. | 1,614 |
| 9 | advanced illness.mp. | 292 |
| 10 | progressive illness.mp. | 105 |
| 11 | life-threatening illness.mp. | 1,197 |
| 12 | 1 or 2 or 3 or 4 or 5 or 6 or 7 or 8 or 9 or 10 or 11 | 30,210 |
| 13 | carer*.mp. | 14,015 |
| 14 | caregiver*.mp. | 84,404 |
| 15 | caregiv*.mp. | 90,791 |
| 16 | family caregiver.mp. | 1,845 |
| 17 | informal care.mp. | 1,697 |
| 18 | unpaid care.mp. | 272 |
| 19 | family care.mp. | 2,098 |
| 20 | lay care.mp. | 28 |
| 21 | elder care.mp. | 7,964 |
| 22 | exp Elder Care/ | 7,594 |
| 23 | home care.mp. | 16,396 |
| 24 | home-based care.mp. | 554 |
| 25 | exp Home Care/ | 8,291 |
| 26 | 13 or 14 or 15 or 16 or 17 or 18 or 19 or 20 or 21 or 22 or 23 or 24 or 25 | 115,936 |
| 27 | support need.mp. | 238 |
| 28 | carer* need.mp. | 89 |
| 29 | supportive care need.mp. | 18 |
| 30 | 27 or 28 or 29 | 344 |
| 31 | need* assessment.mp. | 13,663 |
| 32 | carer* assessment.mp. | 100 |
| 33 | instrument.mp. | 92,220 |
| 34 | carer support needs assessment tool.mp. | 27 |
| 35 | CSNAT.mp. | 15 |
| 36 | instrument development.mp. | 2,041 |
| 37 | 31 or 32 or 33 or 34 or 35 or 36 | 105,250 |
| 38 | 12 and 26 and 30 and 37 | 4 |
| 39 | limit 38 to yr="2024 -Current" | 0 |

**Web of Science Core Collection**

| **#** | **Search query** | **Records** |
| --- | --- | --- |
| 1 | TS=("end of life") | 46,494 |
| 2 | TS=("end-of-life care") | 15,568 |
| 3 | TS=("last year of life") | 891 |
| 4 | TS=(palliative) | 96,566 |
| 5 | TS=("palliative care") | 55,976 |
| 6 | TS=("life limiting") | 3,547 |
| 7 | TS=("terminal illness") | 2,247 |
| 8 | TS=("advanced illness") | 708 |
| 9 | TS=("progressive illness") | 259 |
| 10 | TS=("life-threatening illness") | 1,847 |
| 11 | #1 OR #2 OR #3 OR #4 OR #5 OR #6 OR #7 OR #8 OR #9 OR #10 | 131,387 |
| 12 | TS=(carer*) | 25,016 |
| 13 | TS=(caregiver*) | 124,440 |
| 14 | TS=(caregiv*) | 133,748 |
| 15 | TS=("family caregiver") | 3,238 |
| 16 | TS=("informal care*") | 11,862 |
| 17 | TS=("unpaid care*") | 1,152 |
| 18 | TS=("family care*") | 24,251 |
| 19 | TS=("lay care*") | 232 |
| 20 | TS=("elder care") | 1,576 |
| 21 | TS=("home care") | 22,336 |
| 22 | TS=("home-based care") | 1,464 |
| 23 | #12 OR #13 OR #14 OR #15 OR #16 OR #17 OR #18 OR #19 OR #20 OR #21 OR #22 | 176,192 |
| 24 | TS=("support need*") | 7,168 |
| 25 | TS=("carer* need*") | 411 |
| 26 | TS=("supportive care need*") | 1,808 |
| 27 | #24 OR #25 OR #26 | 9,235 |
| 28 | TS=("need* assessment*") | 13,054 |
| 29 | TS=("carer* assessment*") | 93 |
| 30 | TS=(instrument) | 625,362 |
| 31 | TS=("carer support needs assessment tool") | 44 |
| 32 | TS=(CSNAT) | 75 |
| 33 | TS=("instrument development") | 3,937 |
| 34 | #28 OR #29 OR #30 OR #31 OR #32 OR #33 | 637,706 |
| 35 | #34 AND #27 AND #23 AND #11 | 87 |

**CINAHL (EBSCO)**

end of life or end-of-life care or last year of life or palliative or palliative care or life limiting or terminal illness or advanced illness or progressive illness or life-threatening illness

AND

carer* or caregiver* or caregiv* or family caregiver or informal care* or unpaid care* or family care* or lay care* or elder care or home care or home-based care

AND

support need* or carer* need or supportive care need*

AND

need* assessment* or carer* assessment* or instrument or carer support needs assessment tool or CSNAT or instrument development

English language filter applied.

**ASSIA and ProQuest Central:**

end of life or end-of-life care or last year of life or palliative or palliative care or life limiting or terminal illness or advanced illness or progressive illness or life-threatening illness

AND

carer* or caregiver* or caregiv* or family caregiver or informal care* or unpaid care* or family care* or lay care* or elder care or home care or home-based care

AND

support need* or carer* need or supportive care need*

AND

need* assessment* or carer* assessment* or instrument or carer support needs assessment tool or CSNAT or instrument development

All terms mapped to a subject heading.

**Supplementary Materials 5 – Assessment tools identified, their characteristics and evidence of evaluation**

| **Tool** | **Number of papers using tool** | **Description of tool (taken from original tool development paper where possible)** | **Number and type of evaluation papers** |
| --- | --- | --- | --- |
| Carer Support Needs Assessment Tool (CSNAT/ CSNAT-I) | 35 | Number of items – 14 (later revised to 15)  Domains – 14 (later 15, each item representing one domain) grouped into 2 themes (Enable the carer to care; support for carers)  Who completes – Carer | Validation – 9  Carer feedback – 7  Practitioner feedback – 9  CSNAT adoption – 1  Carer impact - 4 |
| Family Inventory of Needs (FIN) | 6 | Number of items – 20 (each item rated on 2 subscales of importance and fulfilment)  Domains – 1 (unidimensional)  Who completes – Carer | Validation – 3  Carer impact - 1 |
| Carers Alert Thermometer (CAT) | 5 | Number of items – 11*  Domains – 8, grouped into 2 themes (support to provide care and support needed for carer’s own health and wellbeing)  Who completes – Carer  * *1 study provided optional question – end of life care planning.* | Validation – 3  Carer feedback – 1  Practitioner feedback – 1 |
| Spiritual Needs Inventory (SNI) | 4 | Number of items – 17  Domains – 5 (outlook, inspiration, spiritual activities, religion, and community)  Who completes - Carer | Validation – 1  Carer impact - 1 |
| Needs Assessment Tool - Progressive Disease (NAT-PD)*  *Cancer and Heart Failure versions included, plus adapted version for interstitial lung disease | 3 | Number of items – 16  Domains – 0, grouped into 4 themes (Priority for referral to assessment, patients’ wellbeing, ability of carer to care for patient, Carers’/Family’s wellbeing)  Who completes – Clinician (patient/carer informed) | Validation - 3 |
| Home Caregiver Needs Survey (HCNS) | 2 | Number of items - 36  Domains – 6 (information, personal care, psychological support, spiritual support, social support, financial and practical assistance)  Who completes - Carer | Descriptive only |
| Caregiver Strain Index (CSI) | 2 | Number of items – 13  Domains – 0, although sometimes reported in 4 themes (strain in financial, physical, psychological, social and personal)  Who completes - Carer | Descriptive only |
| Preparedness for caregiving scale | 2 | Number of items – 9 (1 is an open-ended written response)  Domains – 0 (unidimensional)  Who completes – Carer | Descriptive only |
| Problems and needs in palliative care questionnaire | 2 | Number of items – 67 items  Domains – 0, grouped into 5 themes (emotional burden and fears, caregiving competence and symptom management, coordination and availability of services, social and role strain, practical and financial problems)  Who completes - carer | Descriptive only |
| Family caregiver in palliative care (CAREPAL-8) | 1 | Number of items - 8  Domains - 0, grouped into 4 themes (psychological distress, unmet needs, reduced quality of life, and limited social support)  Who completes - Carer | Validation - 1 |
| Caregiver Inventory (CGI) (Chinese version) | 1 | Number of items - 18  Domains - 3  Who completes - Carer | Descriptive only |
| Carers Needs Directed Assessment in Dementia (CANDID) | 1 | Number of items - 33  Domains - 0, grouped into 6 assessment themes (symptom management, emergency contacts, advance care planning, carer’s perception of the care recipient's future needs, carer’s current needs, a proposed current and future care plan)  Who completes - Clinician (carer informed) | Carer feedback – 1 |
| Cancer Caregivers Needs Checklist *(developed for a single reporting study)* | 1 | Number of items - 104  Domains - 0, grouped along 10 key experienced during disease trajectory* (start of treatment, end of treatment, non-routine hospitalization, leaving the hospital for home, transitioning from curative to palliative care, entering hospice, patient death, immediately after death, and bereavement)  Who completes - Carers  **10 is the stated number but only nine transition timepoints listed in original reporting.* | Descriptive only |
| Caring Ahead | 1 | Number of items - 20  Domains - 4 (actions, dementia knowledge, communication, emotions and support needs)  Who completes - Carer | Validation - 1 |
| Catholic Healthcare Coalition Family Caregiver Questionnaire | 1 | Number of items – 70 – 81*  Domains – 0, grouped as 4 aspects (physical, spiritual, emotional, relationship)  Who completes - Carer  **stated as a range in the original reporting. We were unable to locate a copy of the instrument to determine precise number for carers.* | Carer Impact - 1 |
| Comprehensive Care Needs Assessment Tool (CNAT-C) | 1 | Number of items: - 41  Domains – 7 (health and psychological problems, family/social support, health-care staff, information, religious/spiritual support, hospital facilities and services, practical support)  Who completes - Carer | Validation - 1 |
| Ongoing Needs Identification: Caregiver Profile Questionnaire (ONI-CP) (Modified version – selected items) | 1 | Number of items - 4  Domains - 0  Who completes - Carer | Descriptive only |
| Carers’ Assessment of Difficulties Index (CADI) (Modified version) | 1 | Number of items: 18  Domains – 0, grouped as 7 subsections (reactions to caring, physical demands of caring, restricted social life, poor family support, poor professional support, financial consequences, carer-dependent relationship).  Who completes - Carer | Descriptive only |
| Unnamed tool from Papadakos et al. (2019) | 1 | Number of items - 55  Domains – 6 (medical, physical, practical, social, emotional, and spiritual informational needs)  Who completes - Carer | Descriptive only |
| Unnamed tool from Thomsen et al. (2017) | 1 | Number of items - 17  Domains - 0  Who completes - Carers | Practitioner feedback - 1 |
| Unnamed tool from Zapata et al. (2022) | 1 | Number of items - 23  Domains – 0, grouped into 4 areas of need for support (knowledge and skills, emotional, health and wellness, practical)  Who completes - Carers | Practitioner feedback – 1  Carer feedback - 1 |
| Unnamed tool from Fukui (2004) | 1 | Number of items - 6  Domains - 0  Who completes - Carers | Descriptive only |
| Unnamed tool from Lin and Tsao (2004) | 1 | Number of items: 105  Domain - 6 (caregiving, disease characteristics, social welfare, psychosocial issues, palliative care, and spirituality and religion)  Who completes - Carers | Validation - 1 |
| Unnamed tool from Wang, et al. (2025) | 1 | Number of items - 34  Domains – 4 (physical harmony, psychological harmony, C: social harmony, spiritual harmony)  Who completes - Carers | Validation - 1 |

*Notes:*

*Tools ordered by study frequency.*

*Descriptive only refers to articles that described carer need but did not evaluate tools used to capture that information.*

*Articles reporting evaluation may have included multiple types and so are counted across multiple categories.*

**Supplementary Materials 6 – Summary of PROGRESS+ characteristics reported in k=64 articles**

*Country of residence*

For country of residence although not all papers reported this explicitly, where authoring teams were based in a single country and/or referenced specific national health care systems, it was clearly evident where the research was undertaken and so the country of residence was coded as having been reported.

The largest number of studies were conducted in the United Kingdom (k=11), Australia (k=10), the United States (k=8), China/Hong Kong (k=6) and Canada (k=5). This was followed by Sweden (k=4), the Netherlands (k=4), Denmark (k=3), Austria (k=3), the Czech Republic (k=2). For Singapore, Japan, Indonesia, Ireland, Taiwan, Turkey, Switzerland and Bhutan, a single study reported findings from each of these as a participant country of residence. Some studies reported data from participants in more than one country.

*Ethnicity/race*

A total of k=22 (34.4%) studies reported some information, on caregivers but what was included was very variably described and reported across the included studies. Terms used varied considerably across studies (e.g. ‘cultural background’, ‘ethnic background’, ‘place of birth’, ‘language spoken’, ‘nationality’) and the same term could be used in different ways (e.g. Australian was reported as both nationality and an ethnic background). Across studies reporting some form of information, Caucasian and non-hispanic white were the largest recorded ethnic categories (18% and 21%).

For care recipients, this information was largely unreported, with data extracted from just k=2 papers.

*Occupation (caregiver)*

Over half of the studies (k=37, 57.8%) provided data relating to the occupation of caregivers. This was usually in the form of simple reporting of employment status (i.e., employed/unemployed/retired; full- or part-time) rather than specific detail about role or occupation class. The clarity and granularity of reporting of occupation status varied between studies, for example, some grouped ‘retired’ and ‘unemployed’ together into a general category that appeared intended to capture non-working status; others grouped ‘retired’ and ‘volunteer’ together into a category that appeared intended to serve as a contrast to that of paid employment.

*Carer gender*

A total of K=56 papers reported the gender of carers with most (k=53) reporting higher proportions of female participants. Where males were in the majority (k=3) this ranged from 51-61% of the sample.

*Care recipient gender*

Of k=21 studies that reported sex/gender of care recipient, k=2 had an equal split between males and females, k=6 reported a majority of female care recipients and the remaining studies (k=13) reported a majority of male care recipients.

*Religion*

Religion was rarely reported (k=8). Of the studies that did obtain religious information, k=4 reported participants were mostly Christian and k=3 reported participants mostly held no religious belief (a further k= 1 study recorded whether participants attended religious confession, with a minority responding they did). Across the k=8 studies reporting religion there were very few participants from other religious categories.

*Education*

Education was recorded in around two-thirds of the papers (k=44) although the level of detail recorded across studies varied with most studies recording level of education obtained but k=5 recording years of education only. Participants were generally well-educated: only k=8 papers reported a majority of participants had not completed secondary education.

*Socio-economic status (income)*

Some means of ascertaining and recording socio-economic status was present in few studies (k=17). This was undertaken variably across studies and included recording income (annual or monthly; individual or household), social security or caregiving compensation (although it was not usually clarified whether the latter was means-tested), and how the caregiving role had impacted employment. Income categories were the most commonly used measure of socio-economic status in the included studies (k=10), with the majority of participants reported as being in the lowest or second-to-lowest group in the majority of studies, but there was little explanation of how income categories and boundaries had been decided upon, or comparison with any relevant (e.g. national) norms.

*Caregiver age*

Caregiver age was presented in different ways across those papers where this information was recorded (k=56). Average age (reported as mean or median) was reported in k=43 papers with an overall average age of 61 years. Age ranges were reported in k=25 papers (k=2 reporting age range only), with the greatest range being 78 years (with participants aged between 19 and 97 years) and the lowest being 21 years (63 to 84 years old). k=12 papers provided summaries of caregivers into different categorised age bands (with k=10 providing only this information on participant age and no further detail).

*Care recipient age*

Patient age was less frequently reported (k=24). Average mean age was reported in k=20 papers, with an overall mean age of 71 years. Age ranges were reported in k=12 papers (k=1 reporting age range only) with the greatest range being 74 years (with care recipients aged between 21 and 95 years) and the lowest being 27 years (42 to 69 years old). Finally, k=5 papers summarised care recipient age into different categorised age bands.

*Relationship between caregiver and care recipient*

The relationship between caregiver and care recipient was reported in k=49 of the studies. The most common relationship (i.e., the relationship status of the highest proportion of participants in the study) was partner/spousal (k=36), followed by adult children (k=13).

*Care recipient’s condition*

Patient condition was reported in k=41 papers. The large majority of studies were conducted only with cancer patients (k=17) or with cancer patients as a majority group amongst other conditions (k=13). Other health conditions of patients included: Motor Neurone Disease, Dementia, various lung and cardiac conditions. Where the patient condition was simply reported as cancer or non-cancer, or does not provide detail on or distinguish between cancer types this tells us little about carer support needs for other, disparately presenting, conditions.

*Carer health condition*

Only k=1 study recorded any specific carer health (long-term/physical health) conditions. A further four studies provided more limited information: k=2 recorded carer self-reported health status within broad categories of response (as excellent, good or fair; as very good, normal, not good enough, poor); k=1 recorded subjective health status of carers, again broadly defined. One k=1 study reported some implied information pertaining to carer health status (whether the carer had sought health care for themselves due to their caring role). A further k=1 study recorded if carers were currently taking leave of absence from employment due to either their caring responsibilities or their own ill health but combined these and did not report separately.

**Supplementary materials 7: Table of Included Articles**

| **Authors** | **Year** | **Country** | **Participants** | **Tool used** |
| --- | --- | --- | --- | --- |
| Alvariza, A., Holm, M., Benkel, I., Norinder, M., Ewing, G., Grande, G., Håkanson, C., Öhlen, J., Årestedt, K. | 2018 | Australia | Informal caregivers | CSNAT |
| Aoun S, Deas K, Toye C, Ewing G, Grande G, Stajduhar K. | 2015 | Australia | Informal caregivers | CSNAT |
| Aoun SM, Deas K, Howting D, Lee G | 2015c | Australia | Informal caregivers | CSNAT |
| Aoun SM, Grande G, Howting D, Deas K, Toye C, Troeung L, et al. | 2015d | Australia | Informal caregivers | CSNAT |
| Aoun SM, Deas K, Kristjanson LJ, Kissane DW | 2017 | Australia | Informal caregivers | CSNAT |
| Aoun SM, Toye C, Slatyer S, Robinson A, Beattie E | 2018 | Australia | Informal caregivers | CSNAT |
| Aoun SM, O'Brien MR, Knighting K. | 2024 | Australia | Informal caregivers | CAT |
| Bijnsdorp FM, Pasman HRW, Boot CRL, van Hooft SM, van Staa A, Francke AL | 2020 | Netherlands | Informal caregivers | Unnamed |
| Brazil K, Kaasalainen S, Williams A, Dumont S | 2014 | Canada | Informal caregivers | HCNS |
| Buck HG, McMillan SC. | 2012 | USA | Informal caregivers | SNI |
| Bužgová R, Špatenková N, Fukasová-Hajnová E, Feltl D | 2016 | Czech Republic | Informal caregivers | FIN |
| Carduff E, Jarvis A, Highet G, Finucane A, Kendall M, Harrison N, Greenacre J, Murray SA | 2016 | UK | Informal caregivers | CSNAT |
| Chan WCH | 2021 | Hong Kong | Informal caregivers | CSNAT |
| Chan WCH, Yu CTK,Kwok DKS, Wan JKM | 2023 | Hong Kong | Informal caregivers | CSNAT  CSI |
| Cheng HL, Leung DYP, Ko PS, Chung MW, Lam WM, Lam PT, Luk AL, Lam SC | 2021 | Hong Kong | Informal caregivers | CSNAT  CSI  Caregiver inventory |
| Cheng HL, Leung DYP, Ko PS, Lam WM, Lam PT, Luk AL, Chung MW, Lam SC | 2023 | Hong Kong | Informal caregivers | CSNAT |
| Du Preez J, Celenza A, Etherton-Beer C, Moffat P, Campbell E, Arendts G | 2023 | Australia | Informal caregivers | CANDID |
| Dubenske LL, Wen KY, Gustafson DH, Guarnaccia CA, Cleary JF, Dinauer SK, McTavish FM | 2008 | USA | Informal caregivers | Cancer Caregivers Needs Checklist |
| Durepos, P., Akhtar-Danesh, N., Sussman, T., Ploeg, J., Boerner, K., Kaasalainen, S | 2021 | Canada | Informal caregivers | Caring Ahead with Dementia questionnaire |
| Ewing G, Brundle C, Payne S, Grande G | 2013 | UK | Informal caregivers | CSNAT |
| Ewing G, Croke S, Rowland C, et al. | 2020 | UK | Informal caregivers | CSNAT |
| Greene A, Aranda S, Tieman JJ, Fazekas B, Currow DC | 2012 | Australia | Informal caregivers | Catholic Healthcare Coalition Family Caregiver Questionnaire  AMA carer self-check |
| Johnson MJ, Jamali A, Ross J, Fairhurst C, Boland J, Reigada C, Hart SP, Grande G, Currow DC, Wells AU, Bajwah S, Papadopoulos T, Bland JM, Yorke J | 2018 | UK | Informal caregivers | NAT:PD |
| Johnston B, Patterson A, Bird L, Wilson E, Almack K, Mathews G, Seymour J. | 2018 | UK | Informal caregivers | CSNAT |
| King C, Khamis A, Ross J, Murtagh FEM, Johnson MJ, Ramsenthaler C | 2022 | UK | Informal caregivers | NAT:PD |
| Knighting K, O'Brien MR, Roe B, Gandy R, Lloyd-Williams M, Nolan M, Jack BA | 2015a | UK | Informal caregivers | CAT |
| Knighting K, O'Brien MR, Roe B, Gandy R, Lloyd-Williams M, Nolan M, Jack BA | 2016 | UK | Informal caregivers | CAT |
| Kreyer C, Bükki J, Pleschberger S | 2020 | Germany, Austria and Switzerland | Informal caregivers | CSNAT |
| Laabar TD, Auret K, Saunders C, Ngo H, Johnson CE | 2022 | Bhutan | Informal caregivers | CSNAT |
| Lund L, Ross L, Petersen MA, Rosted E, Bollig G, Juhl GI, Farholt H, Winther H, Laursen L, Blaaberg EG, Weensgaard S, Guldin MB, Ewing G, Grande G, Groenvold M. | 2022 | Denmark | Informal caregivers | CSNAT |
| Malewezi E, O'Brien M, Knighting K, Thomas J, Jack B | 2023 | UK | Informal caregivers | CAT |
| Marston C, Morgan DD, Philip J, Agar MR | 2023 | Australia | Informal caregivers | CSNAT |
| McIlfatrick S, Doherty LC, Murphy M, Dixon L, Donnelly P, McDonald K, Fitzsimons D | 2018 | UK  Republic of Ireland | Informal caregivers | CSNAT  Preparedness for Caregiving scale |
| McMillan SC, Small BJ, Haley WE. | 2011 | USA | Informal caregivers | SNI |
| Neo SH, Yang GM, Kanesvaran R, Cheung YB. | 2019 | Singapore | Informal caregivers | CNAT-C  FAMCARE |
| Norinder M, Årestedt K, Lind S, Axelsson L, Grande G, Ewing G, Holm M, Öhlén J, Benkel I, Alvariza A. | 2021 | Sweden | Informal caregivers | CSNAT |
| Norinder M, Årestedt K, Axelsson L, Grande G, Ewing G, Alvariza A. | 2024 | Sweden | Informal caregivers | CSNAT |
| Norinder M, Axelsson L, Årestedt K, Grande G, Ewing G, Alvariza A. | 2023 | Sweden | Informal caregivers | CSNAT |
| Osse BH, Vernooij-Dassen MJ, Schadé E, Grol RP | 2006 | Netherlands | Informal caregivers | PNPC |
| Papadakos J, Agarwal A, Charow R, Quartey NK, D'souza A, Giuliani M, Millar BA, Massey C, Shultz D, Chung C | 2019 | Canada | Informal caregivers | Unnamed |
| Pawlow PC, Blumenthal NP, Christie JD, Matura LA, Aryal S, Ersek M | 2021 | USA | Informal caregivers | CSNAT |
| Pidgeon TM, Johnson CE, Lester L, Currow D, Yates P, Allingham SF, Bird S, Eagar K | 2018 | Australia | Informal caregivers | FAMCARE  ONI-CP |
| Purba CIH, Johnston B, Kotronoulas G | 2023 | Indonesia | Informal caregivers | SCNS- P&C |
| Scott G, Whyler N, Grant G. | 2001 | UK | Informal caregivers | CADI |
| Steele RG, Fitch MI | 1996 | Canada | Informal caregivers | HCNS |
| Thomsen KT, Guldin MB, Nielsen MK, Ollars CL, Jensen AB | 2017 | Denmark | Informal caregivers | Risk and needs assessment form |
| Zapata CB, Dionne-Odom JN, Harris HA, Kalanithi L, Lin J, Bischoff K, Fazzalaro K, Pantilat SZ | 2023 | USA | Informal caregivers | Unnamed |
| Zhou S, Zhao Q, Weng H, Wang N, Wu X, Li X, Zhang L | 2021 | China | Informal caregivers | CSNAT |
| Becqué YN, Rietjens JAC, van der Heide A, Witkamp E | 2025 | Netherlands | Informal caregivers | CSNAT |
| Buck, H., McMillan, S.C | 2008 | USA | Informal caregivers | SNI |
| Bužgová R, Kozáková R | 2016 | Czech Republic | Informal caregivers | FIN |
| Fukui S | 2004 | Japan | Informal caregivers | Unnamed |
| Ham L, Fransen HP, de Graeff A, Hendriks MP, de Jong WK, Kloover J, Kuip E, Mandigers C, Sommeijer D, van de Poll L, Raijmakers N, van Zuylen L | 2025 | Netherlands | Informal caregivers | PNPC |
| Huang LT, Tai CY, Longcoy J, McMillan SC. | 2021 | USA | Informal caregivers | SNI |
| Kreyer C, Stecher B, Pleschberger S, Ewing G. | 2024 | Austria | Informal caregivers | CSNAT |
| Kristjanson LJ, Atwood J, Degner LF. | 1995 | USA and Canada | Informal caregivers | FIN |
| Lin WC, Tsao CJ. | 2004 | Taiwan | Informal caregivers | Unnamed |
| Ullrich A, Bergelt C, Marx G, Daubmann A, Benze G, Heine J, Dickel LM, Wowretzko F, Zhang Y, Bokemeyer C, Nauck F, Oechsle K | 2024 | Germany | Informal caregivers | CAREPAL-8 |
| Lund L, Ross L, Petersen MA, Blach A, Rosted E, Bollig G, Juhl GI, Farholt HB, Winther H, Laursen L, Hasse M, Weensgaard S, Guldin MB, Ewing G, Grande G, Groenvold M | 2024 | Denmark | Informal caregivers | CSNAT |
| Sağlam Z, Koç Z. | 2022 | Turkey | Informal caregivers | FIN |
| Schur S, Neubauer M, Amering M, Ebert-Vogel A, Masel EK, Sibitz I, Watzke H, Schrank B | 2015 | Austria | Informal caregivers | FIN |
| Wang L, Li Y, Zhao R, Li H | 2025 | China | Informal caregivers | Unnamed |
| Fridriksdottir N, Sigurdardottir V, Gunnarsdottir S | 2006 | Iceland | Informal caregivers | FIN |
| Aoun S, Toye C, Deas K, Howting D, Ewing G, Grande G, Stajduhar K | 2015b | Australia | Non-carer population (healthcare practitioners) | CSNAT |
| Austin L, Ewing G, Grande G | 2017 | UK | Non-carer population (healthcare practitioners) | CSNAT |
| Diffin J, Ewing G, Harvey G, Grande G | 2018 | UK | Non-carer population (healthcare practitioners) | CSNAT |
| Diffin J, Ewing G, Harvey G, Grande G | 2018b | UK | Non-carer population (healthcare practitioners) | CSNAT |
| Ewing G, Austin L, Diffin J, Grande G | 2015 | UK and Australia | Non-carer population (process paper) | CSNAT |
| Ewing G, Austin L, Grande G | 2016 | UK | Non-carer population (healthcare practitioners) | CSNAT |
| Hall A, Ewing G, Rowland C, Grande G | 2020 | UK | Non-carer population (healthcare practitioners and administrator) | CSNAT |
| Horseman Z, Milton L, Finucane A | 2019 |  | Non-carer population (healthcare practitioners) | CSNAT |
| O’Brien, M., Jack, B., Kinloch, K., Clabburn, O., Knighting, K | 2019 |  | Non-carer population (charity volunteers, charity development advisers and management staff) | CAT |
| Allgar VL, Chen H, Richfield E, Currow D, Macleod U, Johnson MJ | 2018 |  | Non-carer population (healthcare practitioners) | NAT:PD |

**Key**

CSNAT = Carer Support Needs Assessment Tool (CSNAT/CSNAT-I)

CAT = Carers Alert Thermometer

HCNS = Home Carer Needs Survey

SNI = Spiritual Needs Inventory

FIN = Family Inventory of Needs

CSI = Caregiver Strain Index

NAT:PD = Needs Assessment Tool-Progressive Disease

FAMCARE = Family Satisfaction with End-of-Life Care tool (including FAMCARE-2)

PNPC = Problems and Needs in Palliative Care Questionnaire

CANDID = Carer Needs Directed Assessment in Dementia

CNAT-C = Comprehensive Needs Assessment Tool- Caregiver

ONI-CP = Ongoing Needs Identification Profile questionnaire

SCNS- P&C = Supportive Care Needs Survey-Partners and Caregivers

CADI = Carers’ Assessment of Difficulties Index

CAREPAL-8 = 8-item Screening Tool for Family Caregiver Burden in Palliative Care
